# Cross-sectional analysis of the association between personal exposure to household air pollution and blood pressure in adult women: Evidence from the multi-country Household Air Pollution Intervention Network (HAPIN) trial

**DOI:** 10.1016/j.envres.2022.114121

**Published:** 2022-11

**Authors:** Laura Nicolaou, Lindsay Underhill, Shakir Hossen, Suzanne Simkovich, Gurusamy Thangavel, Ghislaine Rosa, John P. McCracken, Victor Davila-Roman, Lisa de las Fuentes, Ashlinn K. Quinn, Maggie Clark, Anaite Diaz, Ajay Pillarisetti, Kyle Steenland, Lance A. Waller, Shirin Jabbarzadeh, Jennifer L. Peel, William Checkley, HAPIN Investigators

**Affiliations:** a Division of Pulmonary and Critical Care, School of Medicine, Johns Hopkins University, Baltimore, USA; b Center for Global Non-Communicable Disease Research and Training, Johns Hopkins University, Baltimore, USA; c Division of Healthcare Delivery Research, MedStar Health Research Institute, Hyattsville, USA; d Division of Pulmonary and Critical Care Medicine, Georgetown University School of Medicine, Washington, USA; e Program in Global Disease Epidemiology and Control, Department of International Health, Bloomberg School of Public Health, Johns Hopkins University, Baltimore, USA; f Sri Ramachandra Institute for Higher Education and Research, Chennai, India; g Faculty of Infectious and Tropical Diseases, London School of Tropical Medicine and Hygiene, London, UK; h Center for Health Studies, Universidad Del Valle de Guatemala, Guatemala City, Guatemala; i Global Health Institute, Epidemiology and Biostatistics Department, University of Georgia, Athens, GA, USA; j Department of Medicine, Washington University in St. Louis, MO, USA; k Environmental Health Sciences, School of Public Health, University of California, Berkeley, CA, USA; l Department of Environmental & Radiological Health Sciences, Colorado State University, Fort Collins, CO, USA; m Department of Environmental Health, Rollins School of Public Health, Emory University, Atlanta, GA, USA; n Department of Biostatistics and Bioinformatics, Rollins School of Public Health, Emory University, Atlanta, GA, USA

**Keywords:** Blood pressure, Household air pollution, Cardiovascular diseases, Low- and middle-income countries

## Abstract

Elevated blood pressure (BP) is a leading risk factor for the global burden of disease. Household air pollution (HAP), resulting from the burning of biomass fuels, may be an important cause of elevated BP in resource-poor communities. We examined the exposure-response relationship of personal exposures to HAP —fine particulate matter (PM_2.5_), carbon monoxide (CO), and black carbon (BC) — with BP measures in women aged 40–79 years across four resource-poor settings in Guatemala, Peru, India and Rwanda. BP was obtained within a day of 24-h personal exposure measurements at baseline, when participants were using biomass for cooking. We used generalized additive models to characterize the shape of the association between BP and HAP, accounting for the interaction of personal exposures and age and adjusting for *a priori* identified confounders. A total of 418 women (mean age 52.2 ± 7.9 years) were included in this analysis. The interquartile range of exposures to PM_2.5_ was 42.9–139.5 μg/m^3^, BC was 6.4–16.1 μg/m^3^, and CO was 0.5–2.9 ppm. Both SBP and PP were positively associated with PM_2.5_ exposure in older aged women, achieving statistical significance around 60 years of age. The exact threshold varied by BP measure and PM_2.5_ exposures being compared. For example, SBP of women aged 65 years was on average 10.8 mm Hg (95% CI 1.0–20.6) higher at 232 μg/m^3^ of PM_2.5_ exposure (90th percentile) when compared to that of women of the same age with personal exposures of 10 μg/m^3^. PP in women aged 65 years was higher for exposures ≥90 μg/m^3^, with mean differences of 6.1 mm Hg (95% CI 1.8–10.5) and 9.2 mm Hg (95% CI 3.3–15.1) at 139 (75th percentile) and 232 μg/m^3^ (90th percentile) respectively, when compared to that of women of the same age with PM_2.5_ exposures of 10 μg/m^3^. Our findings suggest that reducing HAP exposures may help to reduce BP, particularly among older women.

## Highlights

• Household air pollution (HAP) may be a risk factor for elevated blood pressure (BP).
• We examined the link between HAP and BP in women across 4 resource-poor settings.
• Higher BP was associated with higher exposures to PM_2.5_ in women aged >∼60 years.
• Reducing HAP exposures may help to reduce BP, particularly among older women.

## 1 Introduction

High blood pressure is a leading cause of cardiovascular disease and deaths worldwide (Zhou et al., 2021; Nguyen and Chow, 2021; Chow et al., 2013). In 2015, elevated blood pressure was responsible for 8.5 million global deaths from stroke, ischemic heart disease, other vascular diseases, and renal disease, 88% of which occurred in low- and middle-income countries (LMICs) (Zhou et al., 2021; NCD Risk Factor Collaboration, 2021). The number of people with elevated blood pressure was over 1 billion worldwide in 2019 and has doubled since 1990 (Nguyen and Chow, 2021). However, trends in elevated blood pressure prevalence vary globally. While high-income countries have seen a slight decrease in prevalence of elevated blood pressure, LMICs have experienced significant increases in the past two decades (Mills et al., 2016) which threaten to overwhelm healthcare systems, many of which lack effective chronic disease management (Mills et al., 2020). Therefore, identification and mitigation of risk factors for high blood pressure is paramount.

The burning of solid fuels resulting in household air pollution (HAP) is one of the factors that has been associated with elevated blood pressure in LMICs (Murray et al., 2020). HAP is a potentially important attributable risk factor because approximately 3.8 billion people worldwide use solid fuels, including biomass and coal, to cook or heat their homes (Health Effects Institute, 2020). These fuels are often burned in low-efficiency stoves with poor ventilation, resulting in high concentrations of pollutants that are damaging to health including fine particulate matter less than 2.5 μm in aerodynamic diameter (PM_2.5_), carbon monoxide (CO), black carbon (BC), hydrocarbons, nitrogen oxides and sulfur dioxide. Exposure to HAP is associated with endothelial dysfunction, and higher platelet activation, factor VIII levels, oxidative stress and subclinical atherosclerosis (McCracken et al., 2012), all of which are known to adversely affect blood pressure. Epidemiological studies have also shown that ambient PM_2.5_ exposure is associated with elevated blood pressure and adverse CVD outcomes (Du et al., 2016; Yang et al., 2018). However, evidence regarding the association between household PM_2.5_ exposure and elevated blood pressure remains mixed (Khan et al., 2021; Peña et al., 2015; Clark et al., 2013, 2019; Baumgartner et al., 2011; McCracken et al., 2007; Alexander et al., 2015, 2017; Checkley et al., 2021). Reasons for a lack of consistent findings in the association between HAP and BP outcomes may be because previous studies have looked at heterogeneous populations including LMIC individuals who, despite their high exposures to HAP, are mostly healthy. Moreover, data analyses of these studies have mostly ignored potentially important interactions between PM_2.5_ exposures and risk factors for hypertension including age (Harvey et al., 2015) and body mass index (Jiang et al., 2016; Chiang et al., 1969; Kotsis et al., 2010). Indeed, in one observational study conducted in China found that the association between HAP and BP was only positive and significant in women aged >50 years and not in younger women (Clark et al., 2013). Finally, few studies (Zhao et al., 2014; Quinn et al., 2016, 2017) have examined the association of blood pressure (BP) with other pollutants, such as CO and BC.

Using baseline data from the Household Air Pollution Intervention Network (HAPIN) trial, (i.e., after randomization but prior to receiving the intervention), we sought to evaluate the cross-sectional association between BP measures and personal exposures to PM_2.5_, BC and CO in 418 non-pregnant, non-smoking women aged 40–79 years in four resource-poor settings in Guatemala, Peru, India and Rwanda, and identify effect modification by age and body mass index. We used generalized additive models to capture non-linear interactions between continuous variables on blood pressure.

## 2 Methods

### 2.1 Study design and participants

We analyzed cross-sectional data collected at enrollment as part of the HAPIN trial, a randomized controlled trial of a HAP intervention consisting of a liquefied petroleum gas (LPG) stove, continuous fuel distribution and behavioral messaging among 3195 households in four resource-poor LMIC settings: Tamil Nadu, India (T-IND); Jalapa, Guatemala (J-GUA); Puno, Peru (PER); and Kayonza, Rwanda (K-RWA). These four settings were specifically chosen for their low ambient air pollution levels. On average, 24-h PM_2.5_ concentrations were an order of magnitude lower outdoors than in the kitchen, therefore ambient pollution contributed minimally to participants’ personal exposures (Clasen et al., 2020).

The HAPIN trial is described in detail elsewhere (Clasen et al., 2020). Briefly, we enrolled 800 pregnant women aged 18–35 years from households using biomass fuels, and randomized them to intervention or control group on a 1:1 ratio at each study site. We also recruited 418 adult women aged 40–79 years residing in the same households (∼15%) across the sites. These 418 women are the subjects of the present analysis. We used data from the baseline visit in which all 418 households were cooking primarily with biomass stoves. Exclusion criteria included current pregnancy (via self-report), smoking, or planning to permanently move out of the current household in the next 12 months.

The study protocol was reviewed and approved by institutional review boards at Emory University (00089,799), Johns Hopkins University (00007403), Sri Ramachandra Institute of Higher Education and Research (IEC-N1/16/JUL/54/49) and the Indian Council of Medical Research-Health Ministry Screening Committee (5/8/4–30/(Env)/Indo-US/2016-NCD-I), Universidad del Valle de Guatemala (146-08-2016) and Guatemalan Ministry of Health National Ethics Committee (11–2016), Asociación Benefica PRISMA (CE2981.17), the London School of Hygiene and Tropical Medicine (11,664–5) and the Rwandan National Ethics Committee (No.357/RNEC/2018), and Washington University in St. Louis (201611159). The parent trial is registered with ClinicalTrials.gov (Identifier NCT02944682).

### 2.2 Blood pressure measurements

Systolic (SBP) and diastolic blood pressure (DBP) were measured on the right arm in triplicate, with at least 2 min resting between repeat measurements, using an automatic monitor, Omron HEM-907XL (Omron, Tokyo, Japan). The mean of all three measurements was used as the final value in our analyses. Measurements were taken after confirming that the participant had not smoked, consumed alcohol or a caffeinated beverage, or cooked using biomass within 30 min of the measurement. Participants were asked to sit in a chair in a quiet room for 5 min with legs uncrossed, back supported by a chair, and arm supported by a table prior to commencing measurements. Pulse pressure (PP) was calculated as the difference between SBP and DBP, and mean arterial pressure (MAP) was computed as $\frac{1}{3}SBP+\frac{2}{3}DBP$. We used the 2020 International Society of Hypertension Global Hypertension Practice Guidelines to categorize BP (Unger et al., 2020): normal BP for SBP <130 mm Hg and DBP <85 mm Hg; prehypertension for SBP between 130 and 139 mm Hg and/or DBP between 85 and 90 mm Hg; Stage I hypertension for SBP between 140 and 159 mm Hg and/or DBP between 90 and 99 mm Hg; and Stage II hypertension for SBP ≥160 mm Hg and/or DBP ≥100 mm Hg.

### 2.3 Biological and socioeconomic factors

We used a directed acyclic graph to identify confounders of the relationship between BP and HAP (Fig. S1 in the Supplemental Material). Potential risk factors considered were socioeconomic status (SES), body mass index (BMI), second-hand smoking, binge drinking, dietary diversity, physical activity, and a history of diabetes, kidney disease and high cholesterol. Socioeconomic and demographic information for the household were collected through questionnaires during the baseline visit. We surveyed participants about their education, medical history including medication use, tobacco consumption by any household members, physical activity in a typical week, and dietary diversity and alcohol consumption in the last 30 days.

We measured weight and height in duplicate using seca 876 electronic scales and seca 213 stadiometer platforms (seca GmbH & Co. KG., Hamburg, Germany), respectively. If the weight or height measurements differed by more than 0.5 kg or 1 cm, respectively, we collected a third measurement and the two closest readings were averaged. We computed BMI as the ratio between weight and height-squared (kg/m^2^).

We determined dietary diversity using the Minimum Dietary Diversity for Women (MDD-W) indicator, which is based on ten food groups (FAO FHI 360, 2016). Diet diversity was considered low if the participant had consumed less than four of the ten food groups over the previous 30 days (MDD-W < 4), medium if they had consumed four or five food groups (4 ≤ MDD-W ≤ 5), and high if they had consumed more than five (MDD-W > 5). We defined binge drinking as four or more drinks at the same time or within 2 h of each other.

We determined level of physical activity using the Global Physical Activity Questionnaire (GPAQ), which collects information on both vigorous and moderate-intensity activity in three settings: at work, traveling to and from places, and in recreational activities, as well as sedentary behaviour (World Health Organization, 2012). From the GPAQ data, we calculated energy consumption based on Metabolic Equivalents (METs), where one MET is defined as the energy cost of sitting quietly and is equivalent to 1 kcal/kg/hour. Since caloric consumption is an estimated four times as high when moderately active and eight times as high when vigorously active, we calculated overall energy expenditure by assigning four METs to the time spent in moderate activities and eight METs to the time spent in vigorous activities (World Health Organization, 2012).

We used a dimension reduction technique known as principal component analysis (PCA) (Vyas and Kumaranayake, 2006) to construct an SES index based on ownership of selected household assets (n = 24), water and sanitation quality, access to electricity, number of people in the household, food insecurity, participant's education level, and floor, wall and roofing material (Table S1 in the Supplemental Material). We conducted multiple imputation in chain equations for missing data as described elsewhere (Buuren and Groothuis-Oudshoorn, 2011). No variable was missing more than 4% of observations (Fig. S2 in the Supplemental Material). We used the first principal component, which explained 17.9% of the total variance, as our SES index (Houweling et al., 2003).

### 2.4 Personal exposure assessment

We collected personal exposure measurements of PM_2.5_, CO, and black carbon (BC) over a 24-h period at baseline shortly after enrollment. PM_2.5_ exposures were measured using the Enhanced Children's MicroPEM (ECM; RTI Inc, Research Triangle Park, NC, USA), a combined nephelometric and gravimetric PM_2.5_ sampler. The ECM collects PM_2.5_ on a 15-mm diameter filter using a 0.3 L/min pump. We used polytetrafluoroethylene filters with a 2-μm membrane (Measurement Technology Laboratories LLC, Minneapolis, MN, USA) and calibrated the volumetric flowrate of the ECM pumps prior to every sample collection with a TSI 4100 flowmeter (TSI Incorporated, Shoreview, MN, USA) or a Gilibrator-2 primary calibrator (Sensidyne, St Petersburg, FL, USA). Filters were pre- and post-weighed in temperature- and humidity-controlled rooms using 1-μg resolution microbalances (MSA6.6s-000-DF; Sartorius Cubis, Gӧttingen, Germany) at the University of Georgia (filters for Guatemala, Rwanda, and Peru) and at the Sri Ramachandra Institute for Higher Education and Research (for India). We collected four field blanks for every 100 samples and applied median blank corrections by site. Duplicates were collected in a subset of samples to determine monitor performance (Johnson et al., 2021).

Direct-reading CO measurements at 1-min intervals were collected with EL-USB-300 CO monitors (Lascar Electronics, Erie, PA, USA). Calibration checks for the CO monitors were performed every one to three months using a chamber to test the devices with clean air and CO span gas between 40 and 80 ppm (Johnson et al., 2021). Both the EL-USB-CO and ECM are lightweight monitors that can be worn easily without disrupting daily activities. Participants wore these monitors near the breathing zone in pockets of a customized vest or apron provided to them (Johnson et al.,). We asked participants to keep the monitors nearby (within 1–2 m) when sleeping, bathing or conducting other activities for which these could not be safely worn. Daytime wearing compliance was determined based on the ECM accelerometer data, and reported elsewhere (Johnson et al., 2021).

Black carbon deposition on the PM_2.5_ filters was estimated via its black-body optical properties using SootScan OT21 Transmissometers (Magee Scientific, Berkeley, CA, USA), either at the University of Georgia for samples collected in Guatemala, Peru, and Rwanda, or at the Sri Ramachandra Institute for Higher Education and Research for samples collected in India. The instrument measures light attenuation through the filter at the 880-nm wavelength, which is then converted into BC mass deposition using the BC attenuation cross-section from similar source types on similar Teflon filters (Garland et al., 2017).

### 2.5 Biostatistical methods

The primary objective was to model the cross-sectional association between personal exposures to HAP (PM_2.5_, CO, BC) and BP measures (SBP, DBP, MAP, PP) adjusted for *a priori* confounders and accounting for effect modifiers. We used a directed acyclic graph to identify causal and non-causal paths between HAP exposure and BP and identified site and SES as two potential confounders of the relationship between personal exposures to HAP and BP (Fig. S1 in the Supplemental Material). We conducted exposure-response analyses using single-pollutant generalized additive models. Exploratory analyses also revealed that there was an interaction between PM_2.5_ and age on BP measures.

To evaluate the associations between BP outcomes and HAP exposures, we used single-pollutant generalized additive models (GAMs) with tensor-product smooth and cubic regression splines (Hastie and Tibshirani, 2017; Wood, 2006) to model BP as a function of the interaction between HAP and age and adjusted for SES index as a continuous variable and site as a categorical variable. Therefore, the estimated shape of the interaction between HAP and age on BP was a smooth surface that was non-linear in nature. This smooth surface consists of a combination of coefficients and tensor product bases, and the estimated coefficients cannot be interpreted in isolation. An advantage of using a functional data approach for modelling exposure-response relationships is that GAMs can help to uncover complex, non-linear interactions between variables on an outcome. Indeed, a model that included an interaction of HAP and age represented as a smooth surface had the lowest Akaike Information Criterion (AIC) when compared to models with a tensor product smooth spline for HAP only, a tensor product smooth spline for age only, or tensor product smooth splines for both HAP and age (Table S2, Supplemental Material). For SBP and PP, the models with the interaction between PM_2.5_ and age performed best. As such, we used the interaction model consistently across all BP measures and HAP exposures.

**Fig. 1:**
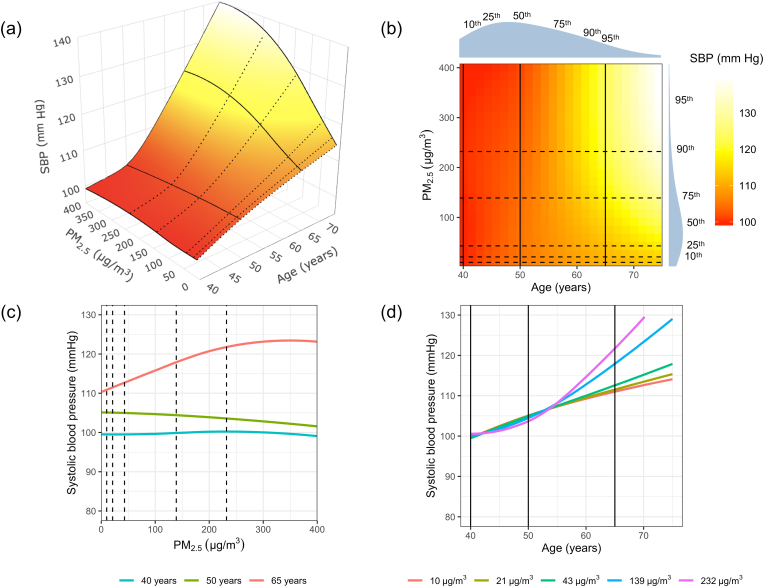
Visual representation of the results of the generalized additive model for systolic blood pressure (SBP) as a three-dimensional smooth surface for the interaction between fine particulate matter (PM_2.5_) and age, adjusted for site and socioeconomic status index using data in 357 women aged 40–79 years living in resource-poor settings of Tamil Nadu, India; Jalapa, Guatemala; Puno, Peru; and Kayonza, Rwanda. Panel (a) plots the estimated three-dimensional surface of SBP as a function of personal exposure to PM_2.5_ and age. The three-dimensional surface is trimmed at the 95th percentile of PM_2.5_ exposures. We used a color gradient from red to white to demonstrate lower to higher values of SBP (in mmHg). The broken lines indicate personal exposures to PM_2.5_ at the 10th, 25th, 75th and 90th percentiles, whereas solid lines indicate age at 40, 50 and 65 years. Panel (b) is a two-dimensional representation of the three-dimensional plot, with the same color gradient from red to white to represent lower to higher values of SBP with lines at values of PM_2.5_ exposures (10th, 25th, 75th, 90th percentile) and age (40, 50, 65 years). The two-dimensional contour plot is also trimmed at the 95th percentile of PM_2.5_ exposures. We also plotted the probability density functions for PM_2.5_ exposures (on the right) and age (on the top) and labelled the location of the 10th, 25th, 75th, 90th and 95th percentiles. In panel (c), we plotted the adjusted associations between SBP and PM_2.5_ exposures at 40, 50 and 65 years of age. In panel (d), we plotted the adjusted associations between SBP and age at PM_2.5_ exposures of 10, 21 (10th percentile), 43 (25th percentile), 139 (75th percentile) and 232 (90th percentile) μg/m^3^. (For interpretation of the references to color in this figure legend, the reader is referred to the Web version of this article.)

In Fig. 1, we show the 3D surface plot (Fig. 1a) and corresponding 2D contour plot (Fig. 1b) of SBP as a function of PM_2.5_ and age. These plots are visually useful to determine whether there are main effects and an interaction present. For example, if there is a positive association between SBP and PM_2.5_ but not with age, the 2D contour plot would only show an increased color gradient from red to white in the vertical axis (i.e., the axis related to the range of PM_2.5_ personal exposure values). If there is a positive association between SBP and age but not with PM_2.5_, then the 2D plot would show an increased color gradient from red to white in the horizontal axis (i.e., the axis related to the range of observed ages). If there are independent associations between SBP and PM_2.5_ and between SBP and age, then the 2D contour plot would show increased color gradients from red to white in both the vertical and horizontal axes, represented by angled, linear stripes. The degree of the angle of the stripes will depend on the relative strength of the association of the two factors. An interaction is likely present when patterns other than those described above are visualized, as seen in Fig. 1b.

The 3D smooth surface function allows us to determine the associations between SBP and PM_2.5_ at any given age, and between SBP and age at any given level of PM_2.5_ exposure. As an example, we're plotting SBP vs PM_2.5_ at three specific ages in Fig. 1c (shown as solid black lines in Fig. 1a), and SBP vs age at five levels of PM_2.5_ exposure in Fig. 1d (shown as dotted black lines in Fig. 1a). In Fig. 1c we observe that the association between PM_2.5_ and SBP differs with age, indicating an interaction between PM_2.5_ and age. Similarly, Fig. 1d shows that the association between age and SBP is different by PM_2.5_ exposure.

We used the estimated models to measure expected differences in BP between PM_2.5_ exposures of either 21, 43, 139 or 232 μg/m^3^ and 10 μg/m^3^, and report the results at three exact ages (40, 50, and 65 years). These concentrations of PM_2.5_ exposures correspond to the 10th, 25th, 75th and 90th percentiles of observed values, and 10 μg/m^3^ was chosen as the lowest interim target (IT-4) recommended by the World Health Organization (WHO) (World Health Organization, 2021). For comparison with the literature, we also examined the mean differences in BP for a 10 μg/m^3^ difference in PM_2.5_ exposure at these three ages. Since the association between BP and PM_2.5_ was non-linear, we evaluated these mean differences at various PM_2.5_ exposures (i.e., 31 vs 21, 53 vs 43, 149 vs 139, and 242 vs 232 μg/m^3^). For BC, we measured expected differences in BP between personal exposures of either 3 (10th percentile), 6 (25th percentile), 16 (75th percentile) or 23 μg/m^3^ (90th percentile) and 1 μg/m^3^ (our chosen reference value). For CO, we measured expected differences in BP between personal exposures of either 0.1 (10th percentile), 0.5 (25th percentile), 2.9 (75th percentile) or 5.8 ppm (90th percentile) and 0 ppm (our chosen reference value). We verified goodness-of-fit of adjusted models by comparing expected and observed BP trajectories with age (Fig. S3 in the Supplemental Material) and HAP exposures (Fig. S4 in the Supplemental Material). Finally, we visually inspected the residual values and found that they followed a normal distribution.

To examine whether BMI was an important effect modifier of the association between BP and HAP, we constructed an additional model with a three-way interaction between HAP exposure, age, and BMI on BP adjusted for site and SES index. As supplementary analysis, we also constructed generalized additive models with age as a categorical variable with three age groups: <50; 50–64; ≥65 years. Further details on the models are provided in the Online Supplement.

We conducted statistical analyses in R version 4.0.4 (Lost Library Book). (R Core Team. R, 2021).

## 3 Results

### 3.1 Descriptive statistics

We summarized baseline characteristics of study participants stratified by study site in Table 1. Data missingness is shown in Fig. S5 (Supplemental Material). Ages ranged between 40.1 and 74.3 years, with a mean (±SD) age of 52.2 ± 7.9 years. There was a difference in the mean BMI of study participants across sites, with mean BMI in the normal range in T-IND (20.9) and K-RWA (23.4), and overweight in J-GUA (25.9) and P-PER (29.0). Most participants in T-IND (96.2%), K-RWA (76.7%) and J-GUA (74.6%) had a low dietary diversity, whereas 81.2% of participants in P-PER had medium or high diet diversity. The SES index was highest in K-RWA and lowest in T-IND, representing lowest and highest SES, respectively (a site-wise summary of variables used in the index is in Table S3 (Supplemental Material) and data missingness is presented in Fig. S1 (Supplemental Material). Exposure to secondhand smoke varied between 0% (P-PER) to 35.6% (T-IND) of participants. The prevalence of self-reported hypertension, diabetes, kidney disease and high cholesterol were below 10% in all sites.

**Table 1:** Characteristics of HAPIN adult women aged 40–79 years at baseline.

		J-GUA	T-IND	P-PER	K-RWA	Overall
		** (n = 138**)	** (n = 104)**	** (n = 133)**	** (n = 43)**	** (n = 418)**
**Mean (SD) or % (n)**						
Systolic blood pressure (mm Hg)		120.8 (21.4)	122.0 (13.9)	107.4 (11.4)	119.0 (17.3)	116.7 (17.6)
Diastolic blood pressure (mm Hg)		71.2 (11.3)	76.7 (9.8)	64.0 (8.7)	73.1 (9.3)	70.5 (11.1)
Pulse pressure (mm Hg)		49.6 (13.2)	45.3 (9.2)	43.4 (7.7)	45.9 (10.7)	46.2 (10.8)
Mean arterial pressure (mm Hg)		87.7 (14.1)	91.8 (10.4)	78.5 (9.0)	88.4 (11.5)	85.9 (12.6)
Blood pressure classification	Normal	76.1% (105)	80.5% (33)	96.1% (123)	67% (69)	80.5% (330)
	Prehypertension	6.5% (9)	9.8% (4)	2.3% (3)	19.4% (20)	8.8% (36)
	Stage I hypertension	10.1% (14)	4.9% (2)	1.6% (2)	12.6% (13)	7.6% (31)
	Stage II hypertension	7.2% (10)	4.9% (2)	0% (0)	1% (1)	3.2% (13)
Age (years)		53.6 (8)	49.1 (6.5)	52.7 (7.9)	53.1 (8.7)	52.2 (7.9)
Weight (kg)		55.1 (9.9)	47.7 (8.4)	65.0 (11.1)	56.1 (10.7)	56.5 (12)
Height (cm)		145.7 (5.4)	151.0 (6.1)	149.8 (4.8)	154.8 (6.5)	149.3 (6.2)
Body mass index (kg/m^2^)		25.9 (4.2)	20.9 (3.3)	28.9 (4.5)	23.4 (4.3)	25.3 (5.1)
Socioeconomic status index (higher is worse)		0.35 (0.65)	−1.11 (0.51)	0.1 (0.52)	1.25 (0.58)	0 (0.91)
Diet diversity	Low	74.6% (103)	96.2% (100)	18.8% (25)	76.7% (33)	62.4% (261)
	Medium	21.7% (30)	3.8% (4)	57.9% (77)	23.3% (10)	28.9% (121)
	High	3.6% (5)	0% (0)	23.3% (31)	0% (0)	8.6% (36)
Physical activity (MET-minutes/week)		3580 (4516)	6905 (5485)	10,223 (7555)	9343 (6173)	7114 (6616)
Binge drinking (days/month)		0 (0.2)	0 (0)	0.1 (0.3)	0.1 (0.3)	0 (0.2)
Second-hand smoke (someone in the household smokes tobacco)		11.6% (16)	35.6% (37)	0% (0)	7% (3)	13.4% (56)
Hypertension medication		7.2% (10)	4.8% (5)	2.3% (3)	9.3% (4)	5.3% (22)
Hypertension (self-reported doctor-diagnosed)		9.4% (13)	0% (0)	4.5% (6)	9.3% (4)	5.5% (23)
Diabetes (self-reported doctor-diagnosed)		3.6% (5)	4.8% (5)	2.3% (3)	2.3% (1)	3.3% (14)
Kidney disease (self-reported doctor-diagnosed)		1.4% (2)	0% (0)	1.5% (2)	11.6% (5)	2.2% (9)
High cholesterol (self-reported doctor-diagnosed)		1.4% (2)	0% (0)	2.3% (3)	0% (0)	1.2% (5)

In our analyses, we excluded seven participants because of missing BP measurements (Fig. S5 in the Supplemental Material) and four because they were pregnant. We also excluded any participants with invalid HAP measurements due to being missing, equipment failure, damaged or misplaced filters, or failure to meet quality assurance criteria (n = 59 for PM_2.5_, n = 103 for BC, n = 46 for CO). A final sample of 357 (85%), 314 (75%), and 362 (87%) participants was included in the PM_2.5_, BC, and CO models, respectively.

### 3.2 Distribution of BP measures

Mean BP varied significantly by site (Table 1). We display the distribution of blood pressure measurements by site in Fig. S6. Mean SBP, DBP and MAP were highest in T-IND (122 ± 14 mm Hg; 77 ± 10 mm Hg; 92 ± 10 mm Hg) and lowest in P-PER (107 ± 11 mm Hg; 64 ± 9 mm Hg; 79 ± 9 mm Hg). Mean PP ranged between 50 ± 13 mm Hg (J-GUA) and 43 ± 8 mm Hg (P-PER). Mean SBP, PP & MAP across all participants increased with age (Fig. S7 in the Supplemental Material). Mean SBP, PP, MAP was higher with larger values of BMI up to ∼26 kg/m^2^ and lower at larger values (Fig. S8; Online Supplement). There was no strong trend in DBP with age or BMI. In all four sites, most participants had normal BP (Table 1; Fig. S9). Average prevalence of normal BP and pre-hypertension was 80.5% (n = 330) and 8.8% (n = 36), respectively. A total of 44 women were identified to have hypertension at baseline, with a prevalence of 10.7% across all sites. J-GUA had the highest percentage of women with hypertension (17.3%) and P-PER the lowest (1.6%).

### 3.3 Distribution of HAP exposures

**Fig. 2:**
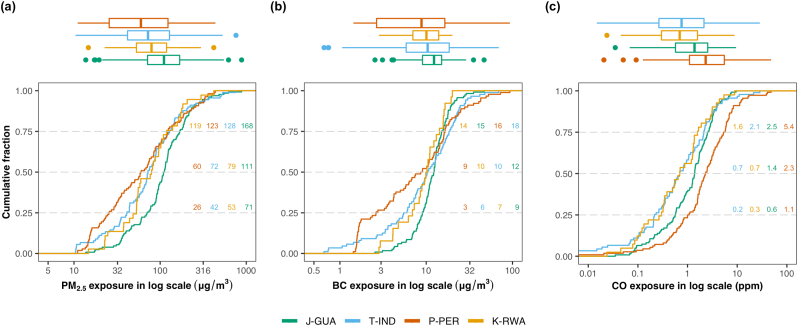
**Distributions of household air pollutants in women aged 40**–**79 years living in resource-poor settings of Tamil Nadu, India; Jalapa, Guatemala; Puno, Peru; and Kayonza, Rwanda.** We plotted cumulative distribution functions and corresponding boxplots of personal exposures to (a) fine particulate matter (PM_2.5_), (b) black carbon (BC), and (c) carbon monoxide (CO) stratified by site, with Jalapa, Guatemala (J-GUA) in green, Tamil Nadu (T-IND) in blue, Puno, Peru (P-PER) in red, and Kayonza, Rwanda (K-RWA) in orange for participants with valid exposure measurements (n = 365, 321 and 371 for PM_2.5_, BC and CO, respectively). Values shown in the cumulative distribution plots represent the first quartile, median, and third quartile at each site. (For interpretation of the references to color in this figure legend, the reader is referred to the Web version of this article.)

We plotted boxplots and cumulative fraction curves of 24-h personal exposures to all three pollutants for all participants aged 40–79 years at baseline and stratified by site in Fig. 2. Median 24-h personal exposures to PM_2.5_ ranged from 60 μg/m^3^ (P-PER) to 111 μg/m^3^ (J-GUA), exceeding the WHO interim air quality target IT-1 of 35 μg/m^3^. The percentage of women with personal exposures below IT-1 was 7.7%, 13.5%, 21.1% and 35.2% in J-GUA, K-RWA, T-IND and P-PER, respectively. On average, BC constituted 14.2 ± 8.9% (mean ± SD) of the PM_2.5_ exposure. Median 24-hr BC exposures were highest in J-GUA (12 μg/m^3^) and lowest in P-PER (9 μg/m^3^), as with PM_2.5_. Personal exposures to CO on the other hand were highest in P-PER, with median 24-hr CO exposures ranging between 0.7 ppm in T-IND and K-RWA and 2.3 ppm in P-PER. Coefficients of variation for PM_2.5_, BC and CO were 0.98, 0.94 and 1.44, respectively.

### 3.4 Associations between household air pollution and blood pressure

**Fig. 3:**
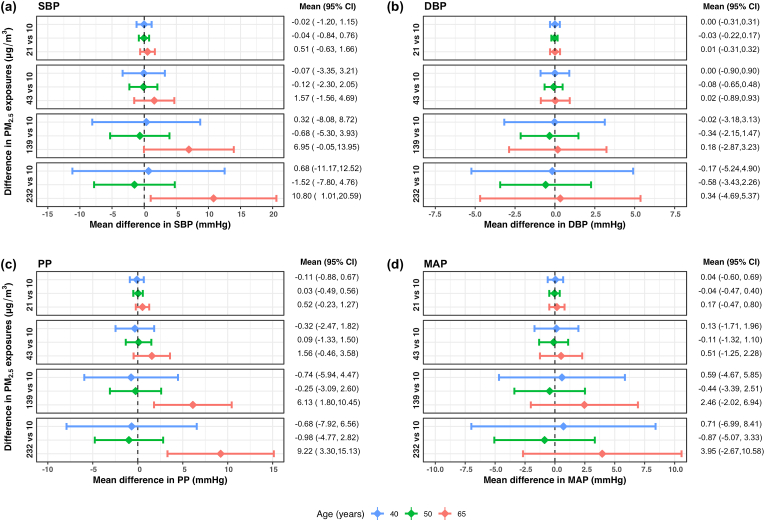
**Estimated mean difference in blood pressure measures between the 10**th**, 25**th**, 75**th **and 90**th **percentile of PM**_**2.5**_**exposures and 10 μg/m**^**3**^**at 40, 50 and 65 years of age using a generalized additive model of blood pressure as a smooth surface of PM**_**2.5**_**exposures and age adjusted for site and socioeconomic status index in 357 women aged 40**–**79 years living in resource-poor settings of Tamil Nadu, India; Jalapa, Guatemala; Puno, Peru; and Kayonza, Rwanda.** Mean differences and 95% pointwise confidence intervals (95% CI) of differences in (a) systolic blood pressure (SBP), (b) diastolic blood pressure (DBP), (c) pulse pressure (PP), and (d) mean arterial pressure (MAP) between PM_2.5_ exposures of either 21, 43, 139 or 232 μg/m^3^ and 10 μg/m^3^ at three specific ages: 40, 50 and 65 years. These PM_2.5_ exposures were chosen based on the 10th, 25th, 75th and 90th percentiles, and 10 μg/m^3^ was chosen as the lowest interim target (IT-4) recommended by the World Health Organization. In each panel, the diamonds represent the mean difference and the horizontal lines represent the corresponding 95% CIs of the mean differences at 40 years (in blue), 50 years (in green), and 65 years (in red). The estimated mean differences (95% CI) in blood pressure measures for differences in PM_2.5_ exposures at the three ages (40, 50, 65 years) are also displayed on the right. (For interpretation of the references to color in this figure legend, the reader is referred to the Web version of this article.)

**Fig. 4:**
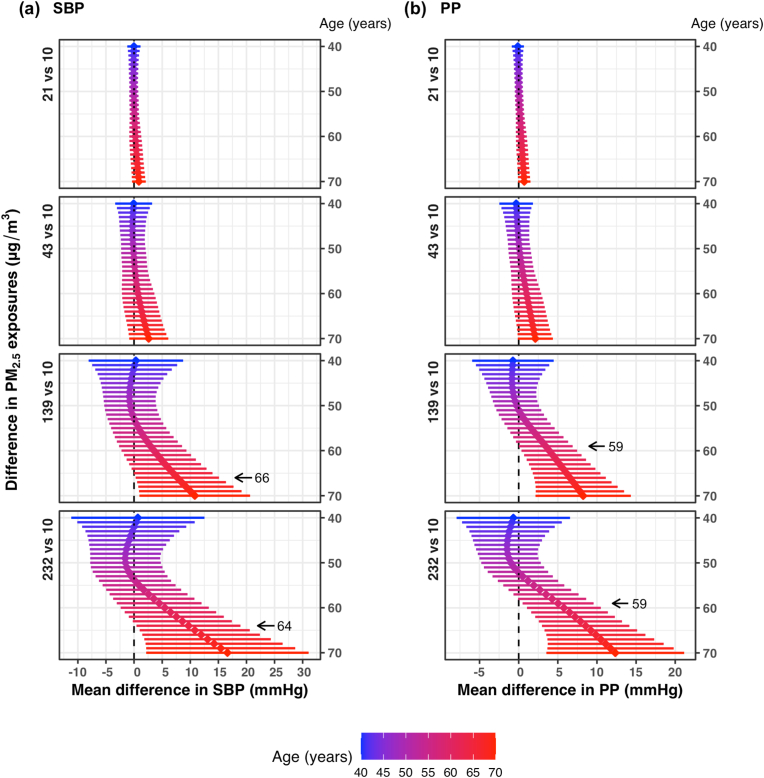
**Estimated mean difference in blood pressure measures between the 10**th**, 25**th**, 75**th **and 90**th **percentile of PM**_**2.5**_**exposures and 10 μg/m**^**3**^**at ages 40 to 70 years using a generalized additive model of blood pressure as a smooth surface of PM**_**2.5**_**exposures and age adjusted for site and socioeconomic status index in 357 women aged 40**–**79 years living in resource-poor settings of Tamil Nadu, India; Jalapa, Guatemala; Puno, Peru; and Kayonza, Rwanda.** Mean differences and 95% pointwise confidence intervals (95% CI) of differences in (a) systolic blood pressure (SBP) and (b) pulse pressure (PP) between PM_2.5_ exposures of either 21, 43, 139 or 232 μg/m^3^ and 10 μg/m^3^ at ages 40–70 years. These PM_2.5_ exposures were chosen based on the 10th, 25th, 75th and 90th percentiles, and 10 μg/m^3^ was chosen as the lowest interim target (IT-4) recommended by the World Health Organization. In each panel, the diamonds represent the mean differences and the horizontal lines represent the corresponding 95% CIs of the mean differences at ages 40–70 years from blue to red. Values shown in the plots represent ages at and above which the mean differences in blood pressure for the given difference in PM_2.5_ exposures become statistically significant. (For interpretation of the references to color in this figure legend, the reader is referred to the Web version of this article.)

We plotted mean differences in BP measures over various levels of personal exposure to PM_2.5_ at 40, 50 and 65 years of age in Fig. 3. The exposure-response curves, at these three ages, are provided in Fig. S10 (Supplemental Material). There were significant mean differences in both SBP and PP at 65 years of age (Fig. 3a,c). On average, SBP at 65 years old was 7.0 mm Hg and 10.8 mm Hg higher at 139 and 232 μg/m^3^ of PM_2.5_, respectively, when compared to that at 10 μg/m^3^. PP at 65 years old was significantly higher for PM_2.5_ exposures ≥90 μg/m^3^ (Fig. S11 in the Supplemental Material), with mean differences of 6.1 mm Hg and 9.2 mm Hg at 139 and 232 μg/m^3^ respectively, compared to that at 10 μg/m^3^. We found no significant differences in DBP or MAP with PM_2.5_ exposure at any age. In Fig. 4, we plotted mean differences in SBP and PP over various levels of personal exposure to PM_2.5_ for all ages from 40 to 70 years. The results show a significant association of PM_2.5_ exposure with SBP and PP in older aged women, achieving statistical significance around 60 years of age and older. The exact threshold varied by BP measure and PM_2.5_ exposures being compared.

**Fig. 5:**
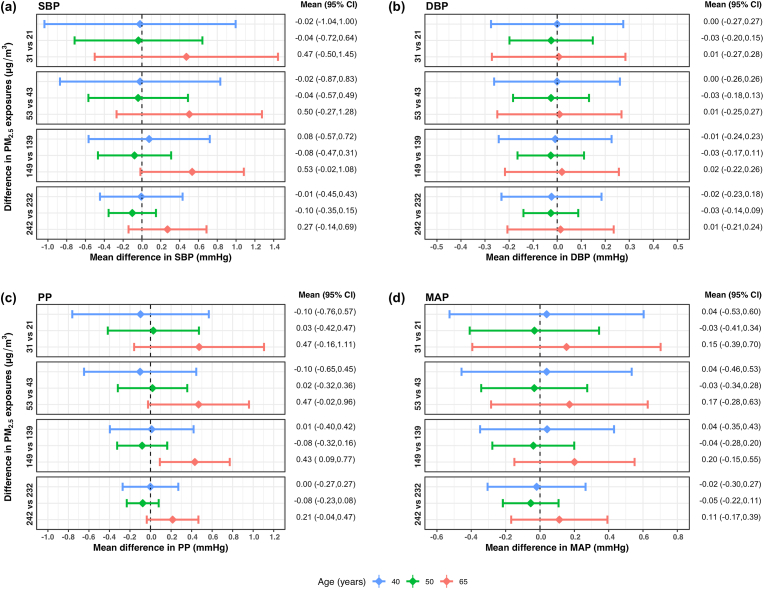
**Estimated mean difference in blood pressure measures between the 10**th**, 25**th**, 75**th **and 90**th **percentile of PM**_**2.5**_**exposures and 10 μg/m**^**3**^**higher at 40, 50 and 65 years of age using a generalized additive model of blood pressure as a smooth surface of PM**_**2.5**_**exposures and age adjusted for site and socioeconomic status index in 357 women aged 40**–**79 years living in resource-poor settings of Tamil Nadu, India; Jalapa, Guatemala; Puno, Peru; and Kayonza, Rwanda.** Mean differences and 95% pointwise confidence interval (95% CI) of differences in (a) systolic blood pressure (SBP), (b) diastolic blood pressure (DBP), (c) pulse pressure (PP), and (d) mean arterial pressure (MAP) between PM_2.5_ exposures of either 21, 43, 139 or 232 μg/m^3^ and 10 μg/m^3^ higher at three specific ages: 40, 50 and 65 years. These PM_2.5_ exposures were chosen based on the 10th, 25th, 75th and 90th percentiles, and 10 μg/m^3^ was chosen as the lowest interim target (IT-4) recommended by the World Health Organization. In each panel, the diamonds represent the mean differences and the corresponding horizontal lines represent the 95% CIs of the mean differences at 40 years (in blue), 50 years (in green), and 65 years (in red). The estimated mean differences (95% CI) in blood pressure measures for differences in PM_2.5_ exposures at the three ages (40, 50, 65 years) are also displayed on the right. (For interpretation of the references to color in this figure legend, the reader is referred to the Web version of this article.)

Mean differences in the four BP outcomes for differences in PM_2.5_ exposure of 10 μg/m^3^ are provided in Fig. 5. The results highlight the non-linear relationship between BP and PM_2.5_ exposure, with mean differences in SBP and PP per 10 μg/m^3^-difference in PM_2.5_ varying depending on the PM_2.5_ level. The largest differences in BP were observed between 149 μg/m^3^ and 139 μg/m^3^, with an increase in SBP of 0.53 (95% pointwise CI -0.02 to 1.08) mm Hg and an increase in PP of 0.43 (95% pointwise CI 0.09 to 0.77) mm Hg at 65 years of age.

We plotted mean differences in our four BP outcomes with differences in BC (Fig. S12 in the Supplemental Material) and CO exposure (Fig. S13 in the Supplemental Material). Similar to PM_2.5_, mean differences in BP varied with age. However, none of the mean differences were significant for BC or CO. The exposure-response curves for our four BP outcomes with BC showed only a weak association between BP and BC exposure at 65 years of age, and no association or weak negative association at ages 40 and 50 years (Fig. S14 in the Supplemental Material). There was no association between BP and CO exposure at any age (Fig. S15 in the Supplemental Material). Results from the models with age as a categorical variable were consistent with these findings (Figs. S16–S18 in the Supplemental Material).

**Fig. 6:**
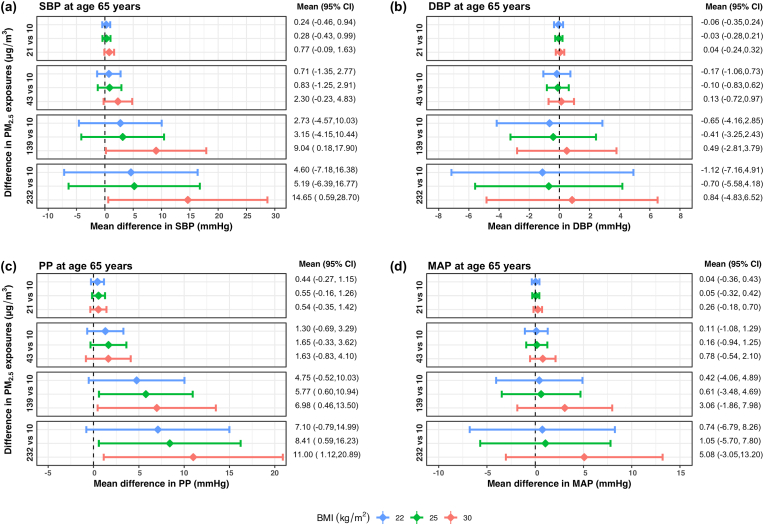
**Effect modification by body mass index (BMI) on the associations between PM**_**2.5**_**exposure and blood pressure measures using a generalized additive model of blood pressure as a smooth surface of PM**_**2.5**_**exposures, age and BMI adjusted for site and socioeconomic status index in 357 women aged 40**–**79 years living in resource-poor settings of Tamil Nadu, India; Jalapa, Guatemala; Puno, Peru; and Kayonza, Rwanda.** Mean differences and 95% pointwise confidence intervals (95% CI) of differences in (a) systolic blood pressure (SBP), (b) diastolic blood pressure (DBP), (c) pulse pressure (PP), and (d) mean arterial pressure (MAP) at 65 years of age, between PM_2.5_ exposures of either 21, 43, 139 or 232 μg/m^3^ and 10 μg/m^3^ at three specific BMIs: 22, 25 and 30 kg/m^2^. These PM_2.5_ exposures were chosen based on the 10th, 25th, 75th and 90th percentiles, and 10 μg/m^3^ was chosen as the lowest interim target (IT-4) recommended by the World Health Organization. In each panel, the diamonds represent the mean differences and the horizontal lines represent the corresponding 95% CIs of the mean differences at a BMI of 22 kg/m^2^ (in blue), 25 kg/m^2^ (in green), and 30 kg/m^2^ (in red). The estimated mean differences (95% CI) in blood pressure measures for differences in PM_2.5_ exposures at the three BMIs (22, 25, 30 kg/m^2^) are also displayed on the right. (For interpretation of the references to color in this figure legend, the reader is referred to the Web version of this article.)

We also plotted the mean differences in BP with differences in PM_2.5_ exposure at 65 years of age and at BMIs of 22, 25 and 30 kg/m^2^ (Fig. 6). The exposure-response curves, at these three BMIs, are provided in Fig. S19 (Supplemental Material). The results show the strong effect modification of BMI on the relationship between PM_2.5_ exposure and BP. On average, the mean difference in SBP between 139 and 10 μg/m^3^ of PM_2.5_ was 6.3 mm Hg higher in women aged 65 years with a BMI of 30 kg/m^2^ (9.0 mm Hg) compared to those with a BMI of 22 kg/m^2^ (2.8 mm Hg). At 232 μg/m^3^, mean differences in SBP compared to that at 10 μg/m^3^ were 4.6 mm Hg, 5.2 mm Hg and 14.7 mm Hg at BMIs of 22, 25 and 30 kg/m^2^, respectively. For PP, mean differences at 139 μg/m^3^ and 232 μg/m^3^ compared to 10 μg/m^3^ were 2.2 mm Hg and 3.9 mm Hg higher, respectively, at a BMI of 30 kg/m^2^ when compared to 22 kg/m^2^. While mean differences in DBP and MAP with higher PM_2.5_ exposures were not significant, similar trends with BMI were also found for these two BP measures.

## 4 Discussion

In this cross-sectional analysis using data from four resource-poor settings, we found that the association between BP and HAP exposure in adult women aged ≥40 years not only varied with age but also with BMI. There was a positive association between PM_2.5_ exposure and BP measures (SBP and PP) that increased linearly and then tapered off in women aged 65 years, whereas there was no relationship between PM_2.5_ and BP measures at 40 or 50 years of age. BMI was also an effect modifier of the relationship between PM_2.5_ exposure and BP. Specifically, when adding BMI in a three-way interaction with PM_2.5_ and age, we found that mean differences in BP with increases in PM_2.5_ exposure at age 65 were greater in participants with a higher BMI.

Findings in the literature regarding the relationship between HAP exposure and BP are mixed (Peña et al., 2015; Clark et al., 2013, 2019; Baumgartner et al., 2011; McCracken et al., 2007; Alexander et al., 2015, 2017; Checkley et al., 2021). While some experimental studies have reported an association between HAP exposure and BP (McCracken et al., 2007; Alexander et al., 2015), others have provided evidence against it (Checkley et al., 2021). Our findings are consistent with previous studies which suggest that HAP exposure is more strongly associated with SBP than DBP (Clark et al., 2013; Baumgartner et al., 2011; Alexander et al., 2015). Previous studies have also reported stronger mean differences with PM_2.5_ exposure among older women (Clark et al., 2013; Baumgartner et al., 2011; Young et al., 2019). This may be attributable to long-term oxidative stress and systemic inflammation due to chronic HAP exposure (Baumgartner et al., 2011). Indeed, aging vessels are thought to have lower anti-oxidant capacity and suffer from more oxidative stress (Csiszar et al., 1985; Zhou et al., 2009) resulting in both impaired endothelial and vasomotor function and an inability to autoregulate BP in the presence of an inflammatory environmental exposure. Therefore, older individuals may experience higher increases in BP when exposed to HAP compared to younger individuals (Bateson and Schwartz, 2004; Goldberg et al., 2001). Effect modification by BMI is also consistent with previous research on HAP (Clark et al., 2013). Indeed, while the mechanisms are not yet well elucidated, obesity may increase susceptibility to the adverse effects of PM_2.5_ on elevated blood pressure and hypertension (Dubowsky et al., 2006; Dong et al., 2015). For example, in a longitudinal cookstove intervention study, the authors observed greater SBP improvements with reductions in indoor PM concentrations among obese compared to non-obese participants (Clark et al., 2013). These findings suggest that older participants and those with a higher BMI may benefit the most from the HAPIN intervention.

We did not find evidence of an exposure-response relationship between BP and CO, which is not consistent with previous findings from Ghana (Quinn et al., 2016). However, this previous study was conducted on pregnant women, which is a different study population from ours. The lack of statistically significant association between CO exposure and BP in our study could also be due to the high variability in CO exposures. While the trend in BP with BC exposure was similar to that with PM_2.5_, mean differences in BP with increases in BC exposure were not statistically significant. A previous study conducted among 205 women in rural China found that among women aged ≥50 years, increased BC exposure was associated with higher SBP, DBP and PP (Baumgartner et al., 2018). It is unclear why our findings differ from these.

Our analysis has several strengths. First, the HAPIN trial was conducted across multiple countries, which helps to provide better generalizability of our findings. Second, we collected a comprehensive set of biological and sociodemographic factors which allowed us to adjust for confounding. Third, we adopted a comprehensive statistical model that captures a non-linear interaction between age and HAP exposure with a smooth surface. Previous exposure-response analyses have not considered two- or three-way interactions between PM_2.5_, age and BMI to describe the relationship with BP measures. Under this modeling framework, coefficients cannot be interpreted in isolation and a linear combination of coefficients are required to construct the estimated exposure-response curves. However, we take full advantage of the continuous nature of the variables and avoid the loss of power that comes with categorization (Royston et al., 2006). Lastly, we evaluated the associations not only with personal PM_2.5_ exposure but also with CO and BC, and examined other BP measures in addition to SBP and DBP not reported in previous HAP studies (PP and MAP) and which have important implications with regards to cardiovascular morbidity and mortality. Indeed, PP, which is an indicator of arterial stiffness, has been shown to be a strong determinant of coronary heart disease, stroke, and cardiovascular events in both hypertensive patients and the general population (Franklin et al., 1999; Benetos et al., 1997). Recent evidence suggests that PP may be a better predictor of coronary heart disease risk than SBP and DBP, especially in older individuals (Cockcroft et al., 2005; Bangalore et al., 2009). Our results, which revealed a stronger association of PM_2.5_ exposure with PP than with other BP measures, suggest that HAP exposure may increase cardiovascular disease risk via an increase in arterial stiffness. Findings from other air pollution studies support this conjecture (Jacobs et al., 2012; Zanoli et al., 2017).

This analysis also has some potential shortcomings. First, we used personal exposures and BP measurements from a single visit rather than multiple visits. Since there can be significant day-to-day variability in HAP exposures, a single measurement may not be representative of a participant's average daily HAP exposures. In addition to day-to-day variability, BP can also vary throughout the day. Future studies may benefit from the use of ambulatory BP monitors that capture BP continuously over a 24-h period. Second, although we adjusted for SES index and site, there may be other unmeasured confounders. Our analysis was also limited to a population of women with low prevalence of self-reported chronic conditions. Therefore, our results cannot be generalized to less healthy populations with underlying conditions such as hypertension or diabetes, or to men. Women, however, tend to have the greatest exposures to HAP as the primary cooks in the household. Finally, while our sample size was larger compared to most previous HAP studies, it may not have been sufficient to detect small mean differences in BP for the range of observed HAP exposures.

In conclusion, we observed a positive exposure-response association between personal exposure to indoor PM_2.5_ and either SBP or PP among women aged around 60 years of age and older, and strong effect modification by BMI. Our findings suggest that reducing HAP exposures may help reduce BP, particularly among older women and those with a higher BMI. Randomized control trials of HAP mitigation like HAPIN are needed to confirm these cross-sectional findings.

## Credit author statement

Laura Nicolaou: Conceptualization, Methodology, Software, Investigation, Formal analysis, Data curation, Writing – original draft, Writing – review & editing, Visualization.; Lindsay Underhill: Project administration, Writing – review & editing.; Shakir Hossen: Data curation, Visualization.; Suzanne Simkovich: Project administration, Writing – review & editing.; Gurusamy Thangavel: Project administration, Resources, Writing – review & editing.; Ghislaine Rosa: Investigation, Project administration, Writing – review & editing.; John P. McCracken: Investigation, Project administration, Resources, Writing – review & editing.; Victor Davila-Roman: Writing – review & editing.; Lisa de las Fuentes: Writing – review & editing.; Ashlinn K. Quinn: Writing – review & editing.; Maggie Clark: Writing – review & editing.; Anaite Diaz: Investigation, Project administration, Resources, Writing – review & editing.; Ajay Pillarisetti: Investigation, Resources, Data curation, Writing – review & editing.; Kyle Steenland: Investigation, Writing – review & editing.; Lance A. Waller: Data curation, Writing – review & editing.; Shirin Jabbarzadeh: Data curation, Writing – review & editing.; Jennifer L. Peel: Investigation, Writing – review & editing, Supervision, Project administration, Funding acquisition.; William Checkley: Conceptualization, Methodology, Investigation, Writing – review & editing, Supervision, Project administration, Visualization, Funding acquisition.

## Declaration of competing interest

The authors declare that they have no known competing financial interests or personal relationships that could have appeared to influence the work reported in this paper.

## Data Availability

Data will be made available on request.
