# Supplementary material for: Cross-sectional analysis of the association between personal exposure to household air pollution and blood pressure in adult women: Evidence from the multi-country Household Air Pollution Intervention Network (HAPIN) trial
Source: Environ Res. 2022 Nov;214:114121. doi: 10.1016/j.envres.2022.114121 (PMC9492861; doi:10.1016/j.envres.2022.114121)
Supplement: Multimedia component 1 [file mmc1.docx]

**Supplemental Material**

**Cross-sectional analysis of the association between personal exposure to household air pollution and blood pressure in adult women: Evidence from the multi-country Household Air Pollution Intervention Network (HAPIN) trial**

Laura Nicolaou, Lindsay Underhill, Shakir Hossen, Suzanne Simkovich, Gurusamy Thangavel, Ghislaine Rosa, John McCracken, Victor Davila-Roman, Lisa de las Fuentes, Ashlinn K. Quinn, Maggie Clark, Anaite Diaz, Ajay Pillarisetti, Kyle Steenland, Lance Waller, Shirin Jabbarzadeh, Jennifer L. Peel, William Checkley on behalf of HAPIN Investigators

S1. Biostatistical methods

Table S1: Variables used in the principal component analysis construction of the SES index.

Table S2: Model formula and Akaike Information Criterion (AIC) used in model selection.

Table S3: SES characteristics of HAPIN adult women aged 40 – 79 years by site and overall.

Figure S1: Directed acyclic graph representing the causal assumptions used and the minimal adjustment set to avoid confounding.

Figure S2: Missingness plot for SES variables used in SES index.

Figure S3: Goodness of fit: Mean and 95% confidence intervals (95% CIs) of expected and observed blood pressure trajectories with age.

Figure S4: Goodness of fit: Mean and 95% confidence intervals (95% CIs) of expected and observed blood pressure trajectories with exposures to household air pollutants.

Figure S5: Missingness plot for baseline variables.

Figure S6: Distributions of blood pressure measures by site: Cumulative distribution functions and corresponding boxplots of (a) systolic blood pressure, (b) diastolic blood pressure, (c) pulse pressure, and (d) mean arterial pressure.

Figure S7: Mean and 95% confidence intervals (95% CIs) of blood pressure measures with age, modeled using natural splines with three degrees of freedom.

Figure S8: Mean and 95% confidence intervals (95% CIs) of blood pressure measures with body mass index (BMI), modeled using natural splines with three degrees of freedom.

Figure S9: Blood pressure classification of all participants aged 40 – 79 years stratified by site.

Figure S10: Associations between blood pressure measures and fine particulate matter (PM_2.5_) exposures at 40, 50 and 65 years of age using a generalized additive model of blood pressure as a smooth surface of PM_2.5_ exposures and age adjusted for site and socioeconomic status index in 357 women aged 40-79 years living in resource-poor settings of Tamil Nadu, India; Jalapa, Guatemala; Puno, Peru; and Kayonza, Rwanda.

Figure S11: Estimated mean difference in blood pressure measures between fine particulate matter (PM_2.5_) exposures of 20 to 250 μg/m^3^ and 10 μg/m^3^ at 40, 50 and 65 years of age using a generalized additive model of blood pressure as a smooth surface of PM_2.5_ exposures and age adjusted for site and socioeconomic status index in 357 women aged 40-79 years living in resource-poor settings of Tamil Nadu, India; Jalapa, Guatemala; Puno, Peru; and Kayonza, Rwanda.

Figure S12: Estimated mean difference in blood pressure measures between black carbon (BC) exposures of either 3, 6, 16 or 23 μg/m^3^ and 1 μg/m^3^ at 40, 50 and 65 years of age using a generalized additive model of blood pressure as a smooth surface of BC exposures and age adjusted for site and socioeconomic status index in 314 women aged 40-79 years living in resource-poor settings of Tamil Nadu, India; Jalapa, Guatemala; Puno, Peru; and Kayonza, Rwanda.

Figure S13: Estimated mean difference in blood pressure measures between carbon monoxide (CO) exposures of either 0.1, 0.5, 2.9 or 5.8 ppm and 0 ppm at 40, 50 and 65 years of age using a generalized additive model of blood pressure as a smooth surface of CO exposures and age adjusted for site and socioeconomic status index in 362 women aged 40-79 years living in resource-poor settings of Tamil Nadu, India; Jalapa, Guatemala; Puno, Peru; and Kayonza, Rwanda.

Figure S14: Associations between blood pressure measures and black carbon (BC) exposures at 40, 50 and 65 years of age using a generalized additive model of blood pressure as a smooth surface of BC exposures and age adjusted for site and socioeconomic status index in 314 women aged 40-79 years living in resource-poor settings of Tamil Nadu, India; Jalapa, Guatemala; Puno, Peru; and Kayonza, Rwanda.

Figure S15: Associations between blood pressure measures and carbon monoxide (CO) exposures at 40, 50 and 65 years of age using a generalized additive model of blood pressure as a smooth surface of CO exposures and age adjusted for site and socioeconomic status index in 362 women aged 40-79 years living in resource-poor settings of Tamil Nadu, India; Jalapa, Guatemala; Puno, Peru; and Kayonza, Rwanda.

Figure S16: Estimated mean difference in blood pressure measures between fine particulate matter (PM_2.5_) exposures of either 21, 43, 139 or 232 μg/m^3^ and 10 μg/m^3^ for ages <50; 50-64; ≥65 years using a generalized additive model of blood pressure as a spline of PM_2.5_ exposures by age group adjusted for site and socioeconomic status index in 357 women aged 40-79 years living in resource-poor settings of Tamil Nadu, India; Jalapa, Guatemala; Puno, Peru; and Kayonza, Rwanda.

Figure S17: Estimated mean difference in blood pressure measures between black carbon (BC) exposures of either 3, 6, 16 or 23 μg/m^3^ and 1 μg/m^3^ for ages <50; 50-64; ≥65 years using a generalized additive model of blood pressure as a spline of BC exposures by age group adjusted for site and socioeconomic status index in 314 women aged 40-79 years living in resource-poor settings of Tamil Nadu, India; Jalapa, Guatemala; Puno, Peru; and Kayonza, Rwanda.

Figure S18: Estimated mean difference in blood pressure measures between carbon monoxide (CO) exposures of either 0.1, 0.5, 2.9 or 5.8 ppm and 0 ppm for ages <50; 50-64; ≥65 years using a generalized additive model of blood pressure as a spline of CO exposures by age group adjusted for site and socioeconomic status index in 362 women aged 40-79 years living in resource-poor settings of Tamil Nadu, India; Jalapa, Guatemala; Puno, Peru; and Kayonza, Rwanda.

Figure S19: Associations between blood pressure measures and fine particulate matter (PM_2.5_) exposures at 65 years of age for BMIs of 22, 25 and 30 kg/m^2^ using a generalized additive model of blood pressure as a smooth surface of PM2.5 exposures, age and BMI, adjusted for site and socioeconomic status index in 357 women aged 40-79 years living in resource-poor settings of Tamil Nadu, India; Jalapa, Guatemala; Puno, Peru; and Kayonza, Rwanda.

**S1. Biostatistical methods**

We used generalized additive models (GAMs) to estimate the difference in blood pressure outcomes (SBP, DBP, MAP, PP) as a function of personal exposure to HAP (PM_2.5_, CO, BC). We used a tensor-product smooth with cubic regression splines to model the nonlinear interaction between age and personal exposure. Our model is given by:

$y_{i}=\beta_{0}+\sum_{k=1}^{3} \beta_{k}I_{k}+\beta_{4}w_{i}+ te\left( x_{i},t_{i} \right)+\varepsilon_{ij}$,

where $y_{i}$ is the BP outcome (SBP, DBP, MAP or PP), $I_{k}$ are the indicator variables for sites, $w_{i.}$is the wealth index, $x_{i}$ is the personal exposure concentration (PM_2.5_, CO, or BC), $t_{i}$ is age and $te\left( \cdot\right)$ is a tensor-product spline.

To examine the effect modification by BMI, $m_{i}$, we adopted the following model:

$y_{i}=\beta_{0}+\sum_{k=1}^{3} \beta_{k}I_{k}+\beta_{4}w_{i}+ te\left( x_{i},t_{i},m_{i} \right)+\varepsilon_{ij}$.

As supplementary analysis, we also constructed generalized additive models with age as a categorical variable with three age groups: ≤50; 51-65; >65 years:

$$y_{i}=\beta_{0}+\sum_{k=1}^{3} \beta_{k}I_{k}+\beta_{4}w_{i}+ f_{i}\left( x_{i};T_{i} \right)+\varepsilon_{ij},$$

where $f_{i}\left( \cdot\right)$ is a thin-plate spline and $T_{i}$ is the age group.

**Table S1: Variables used in the principal component analysis construction of the SES index.**

| **Variable** | | **Type** | **Values** | | | |
| --- | --- | --- | --- | --- | --- | --- |
| Roof material | | Dichotomous | **0 = Unimproved**  thatch  reed  wattle  mud | | **1 = Improved**  mud brick  earthen tile stone  fired brick  corrugated metal  corrugated fiberglass  concrete/cement  wood  vinyl  fired tile | |
| Wall material | | Dichotomous | **0 = Unimproved**  thatch  reed  wattle  mud | | **1 = Improved**  mud brick  earthen tile stone  fired brick  corrugated metal  corrugated fiberglass  concrete/cement  wood  vinyl  fired tile | |
| Floor material | | Dichotomous | **0 = Unimproved**  thatch  reed  wattle  mud | | **1 = Improved**  mud brick  earthen tile stone  fired brick  corrugated metal  corrugated fiberglass  concrete/cement  wood  vinyl  fired tile | |
| Household assets | Color television | Dichotomous | **0 = No** | | **1 = Yes** | |
|  | Cable/dish television | Dichotomous | **0 = No** | | **1 = Yes** | |
|  | Radio | Dichotomous | **0 = No** | | **1 = Yes** | |
|  | Computer | Dichotomous | **0 = No** | | **1 = Yes** | |
|  | Internet | Dichotomous | **0 = No** | | **1 = Yes** | |
|  | Mobile phone | Dichotomous | **0 = No** | | **1 = Yes** | |
|  | Wrist watch | Dichotomous | **0 = No** | | **1 = Yes** | |
|  | Air cooler/air conditioner | Dichotomous | **0 = No** | | **1 = Yes** | |
|  | Space heater | Dichotomous | **0 = No** | | **1 = Yes** | |
|  | Bookshelf | Dichotomous | **0 = No** | | **1 = Yes** | |
|  | Windows with cloth curtains/blinds | Dichotomous | **0 = No** | | **1 = Yes** | |
|  | Sofa | Dichotomous | **0 = No** | | **1 = Yes** | |
|  | Dining room table | Dichotomous | **0 = No** | | **1 = Yes** | |
|  | Mattress | Dichotomous | **0 = No** | | **1 = Yes** | |
|  | Microwave | Dichotomous | **0 = No** | | **1 = Yes** | |
|  | Pressure cooker | Dichotomous | **0 = No** | | **1 = Yes** | |
|  | Blender | Dichotomous | **0 = No** | | **1 = Yes** | |
|  | Refrigerator | Dichotomous | **0 = No** | | **1 = Yes** | |
|  | Bank account | Dichotomous | **0 = No** | | **1 = Yes** | |
|  | Clothes washing machine | Dichotomous | **0 = No** | | **1 = Yes** | |
|  | Bicycle | Dichotomous | **0 = No** | | **1 = Yes** | |
|  | Motorcycle/scooter | Dichotomous | **0 = No** | | **1 = Yes** | |
|  | Car or truck | Dichotomous | **0 = No** | | **1 = Yes** | |
|  | Tractor/large farming equipment | Dichotomous | **0 = No** | | **1 = Yes** | |
| Electricity | | Dichotomous | **0 = No** | | **1 = Yes** | |
| Water source | | Dichotomous | **0 = Unimproved**  Unprotected dug well  Unprotected spring  Surface water | | **1 = Improved**  Piped water into dwelling  Piped water to yard/plot  Piped water to neighbor  Piped water-Public tap/standpipe Tube Well/Borehole  Protected dug well  Protected spring  Rainwater  Tanker-truck  Cart with small tank  Water kiosk  Packaged water: Bottled water  Packaged water: Sachet water | |
| Sanitation | | Dichotomous | **0 = Unimproved**  No facility/Bush/Field  Flush/pour to open drain  Flush/pour to unknown location  Pit latrine without slab/open pit  Twin pit without slab  Bucket  Hanging toilet/Hanging latrine | | **1 = Improved**  Flush/pour to piped sewer  Flush/pour to septic tank  Flush/pour to pit latrine  Pit latrine with slab  Composting toilet  Twin pit with slab | |
| Number of people sleeping in household | | Continuous | **Reciprocal**  (smaller value corresponds to lower SES) | | | |
| Food insecurity | | Categorical | **1 = Moderate/Severe** | **2 = Mild** | | **3 = None** |
| Education | | Categorical | **1 = No formal education or Primary school incomplete** | **2 = Primary school complete or Secondary school incomplete** | | **3 = Secondary school complete or Vocational or Some college or university** |

**Table S2: Model formulae and Akaike Information Criterion (AIC) values used for model selection.**

| **BP measure** |  | **Model 1** | **Model 2** | **Model 3** | **Model 4** | **Model 5** |
| --- | --- | --- | --- | --- | --- | --- |
| **SBP** | **Formula** | SBP ~ 1 | SBP ~ ti(pm) | SBP ~ ti(age) | SBP ~ ti(pm) + ti(age) | SBP ~ ti(pm) + ti(age) + ti(pm, age) |
|  | **AIC** | 3087.87 | 3089.321 | 3041.151 | 3041.496 | 3038.616 |
| **DBP** | **Formula** | DBP ~ 1 | DBP ~ ti(pm) | DBP ~ ti(age) | DBP ~ ti(pm) + ti(age) | DBP ~ ti(pm) + ti(age) + ti(pm, age) |
|  | **AIC** | 2745.91 | 2748.162 | 2727.465 | 2729.736 | 2731.856 |
| **PP** | **Formula** | PP ~ 1 | PP ~ ti(pm) | PP ~ ti(age) | PP ~ ti(pm) + ti(age) | PP ~ ti(pm) + ti(age) + ti(pm, age) |
|  | **AIC** | 2728.379 | 2729.799 | 2633.611 | 2632.668 | 2624.913 |
| **MAP** | **Formula** | MAP ~ 1 | MAP ~ ti(pm) | MAP ~ ti(age) | MAP ~ ti(pm) + ti(age) | MAP ~ ti(pm) + ti(age) + ti(pm, age) |
|  | **AIC** | 2846.053 | 2848.15 | 2821.675 | 2823.422 | 2823.626 |

**Table S3: SES characteristics of HAPIN adult women aged 40 – 79 years by site and overall.**

|  | | **J-GUA** | **T-IND** | **P-PER** | **K-RWA** | **Overall** |
| --- | --- | --- | --- | --- | --- | --- |
|  | | **(n = 138**) | **(n = 104)** | **(n= 133)** | **(n = 43)** | **(n = 418)** |
| **Mean (SD) or % (n)** | |  |  |  |  |  |
| Improved Roof | | 100% (138) | 57.7% (60) | 98.5% (131) | 100% (43) | 89% (372) |
| Improved Floor | | 35.5% (49) | 58.7% (61) | 54.9% (73) | 30.2% (13) | 46.9% (196) |
| Improved Wall | | 98.6% (136) | 57.7% (60) | 98.5% (131) | 100% (43) | 88.5% (370) |
| TV | | 52.2% (72) | 75% (78) | 57.1% (76) | 9.3% (4) | 55% (230) |
| Cable TV | | 34.8% (48) | 69.2% (72) | 7.5% (10) | 2.3% (1) | 31.3% (131) |
| Radio | | 46.4% (64) | 18.3% (19) | 78.9% (105) | 46.5% (20) | 49.8% (208) |
| Computer | | 2.9% (4) | 9.6% (10) | 3% (4) | 0% (0) | 4.3% (18) |
| Internet | | 0% (0) | 37.5% (39) | 0.8% (1) | 0% (0) | 9.6% (40) |
| Cell phone | | 97.1% (134) | 80.8% (84) | 98.5% (131) | 74.4% (32) | 91.1% (381) |
| Wrist watch | | 15.9% (22) | 41.3% (43) | 14.3% (19) | 32.6% (14) | 23.4% (98) |
| Air cooler/air conditioner | | 0% (0) | 0% (0) | 0% (0) | 2.3% (1) | 0.2% (1) |
| Space heater | | 0.7% (1) | 1% (1) | 0% (0) | 0% (0) | 0.5% (2) |
| Bookshelf | | 2.9% (4) | 10.6% (11) | 16.5% (22) | 2.3% (1) | 9.1% (38) |
| Blind | | 2.2% (3) | 4.8% (5) | 27.1% (36) | 37.2% (16) | 14.4% (60) |
| Sofa | | 3.6% (5) | 7.7% (8) | 6.8% (9) | 14% (6) | 6.7% (28) |
| Table | | 32.6% (45) | 0% (0) | 20.3% (27) | 81.4% (35) | 25.6% (107) |
| Mattress | | 80.4% (111) | 42.3% (44) | 86.5% (115) | 88.4% (38) | 73.7% (308) |
| Microwave | | 4.3% (6) | 1% (1) | 0% (0) | 0% (0) | 1.7% (7) |
| Cooker | | 1.4% (2) | 17.3% (18) | 27.8% (37) | 2.3% (1) | 13.9% (58) |
| Blender | | 23.9% (33) | 88.5% (92) | 35.3% (47) | 0% (0) | 41.1% (172) |
| Refrigerator | | 14.5% (20) | 9.6% (10) | 6% (8) | 2.3% (1) | 9.3% (39) |
| Bank account | | 31.9% (44) | 89.4% (93) | 28.6% (38) | 23.3% (10) | 44.3% (185) |
| Washing machine | | 0% (0) | 1% (1) | 0.8% (1) | 0% (0) | 0.5% (2) |
| Bicycle | | 14.5% (20) | 13.5% (14) | 49.6% (66) | 27.9% (12) | 26.8% (112) |
| Motorcycle/scooter | | 15.9% (22) | 77.9% (81) | 72.9% (97) | 0% (0) | 47.8% (200) |
| Car or truck | | 3.6% (5) | 1.9% (2) | 9.8% (13) | 0% (0) | 4.8% (20) |
| Tractor | | 0.7% (1) | 2.9% (3) | 2.3% (3) | 0% (0) | 1.7% (7) |
| Electricity | | 94.9% (131) | 97.1% (101) | 94% (125) | 27.9% (12) | 88.3% (369) |
| Improved water source | | 87% (120) | 81.7% (85) | 81.2% (108) | 81.4% (35) | 83.3% (348) |
| Improved sanitation | | 96.4% (133) | 23.1% (24) | 31.6% (42) | 90.7% (39) | 56.9% (238) |
| People sleeping in household | | 7.72 (2.91) | 4.37 (1.34) | 5.6 (1.82) | 5.93 (2.15) | 6.03 (2.54) |
| Food insecurity | Moderate/severe | 10.9% (15) | 3.8% (4) | 14.3% (19) | 44.2% (19) | 13.6% (57) |
|  | Mild | 33.3% (46) | 16.3% (17) | 39.1% (52) | 27.9% (12) | 30.4% (127) |
|  | None | 53.6% (74) | 79.8% (83) | 44.4% (59) | 27.9% (12) | 54.5% (228) |
| Education | < Primary | 91.3% (126) | 95.2% (99) | 60.2% (80) | 69.8% (30) | 80.1% (335) |
|  | Primary-Secondary | 2.9% (4) | 4.8% (5) | 36.8% (49) | 16.3% (7) | 15.6% (65) |
|  | ≥ Secondary | 0.7% (1) | 0% (0) | 2.3% (3) | 14% (6) | 2.4% (10) |

**Figure S1: Directed acyclic graph representing the causal assumptions used and the minimal adjustment set to avoid confounding.**

**
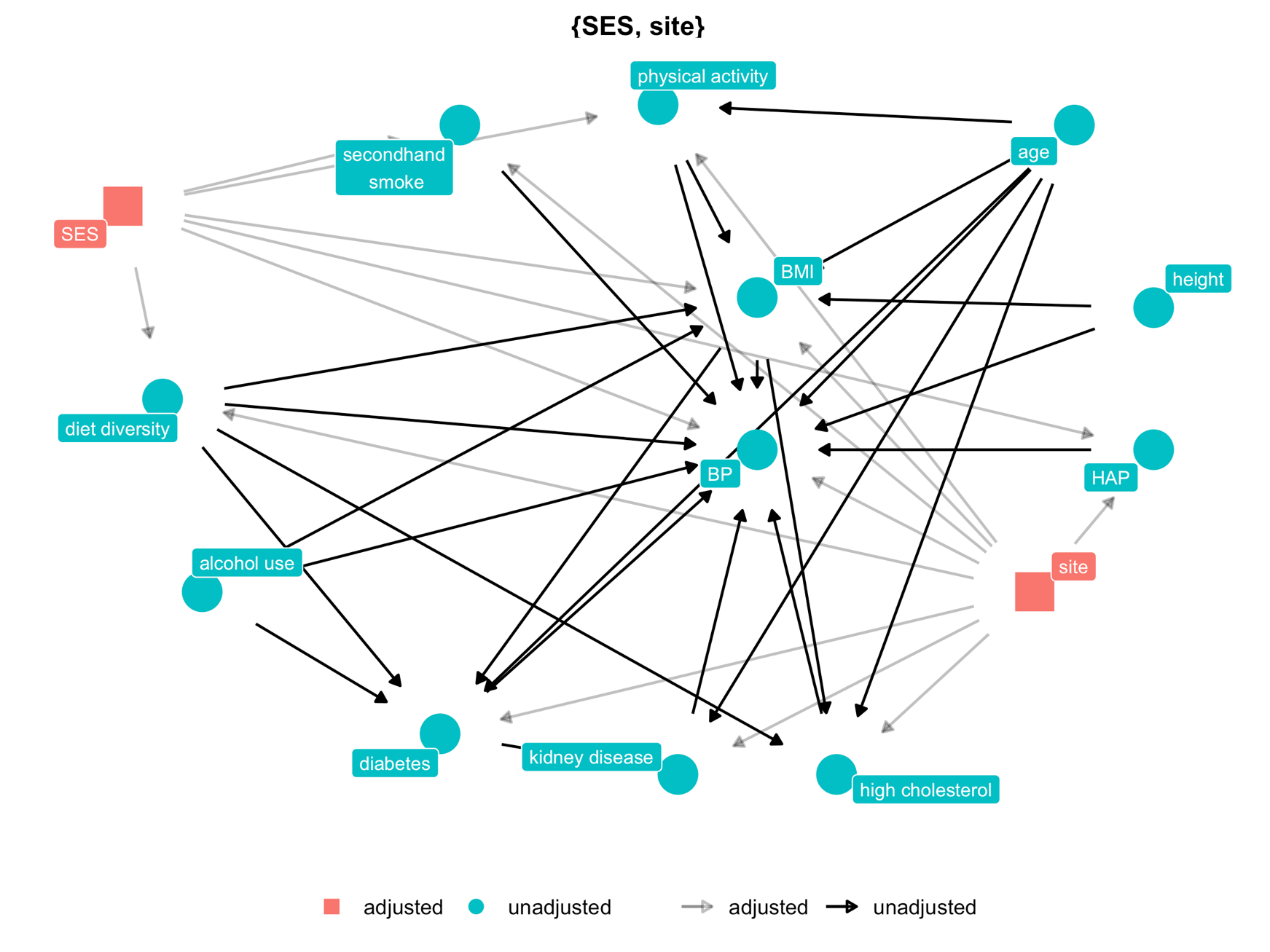
**

**Figure S2: Missingness plot for SES variables used in SES index.** Values displayed on the right represent the number (percentage) missing for each variable.

**
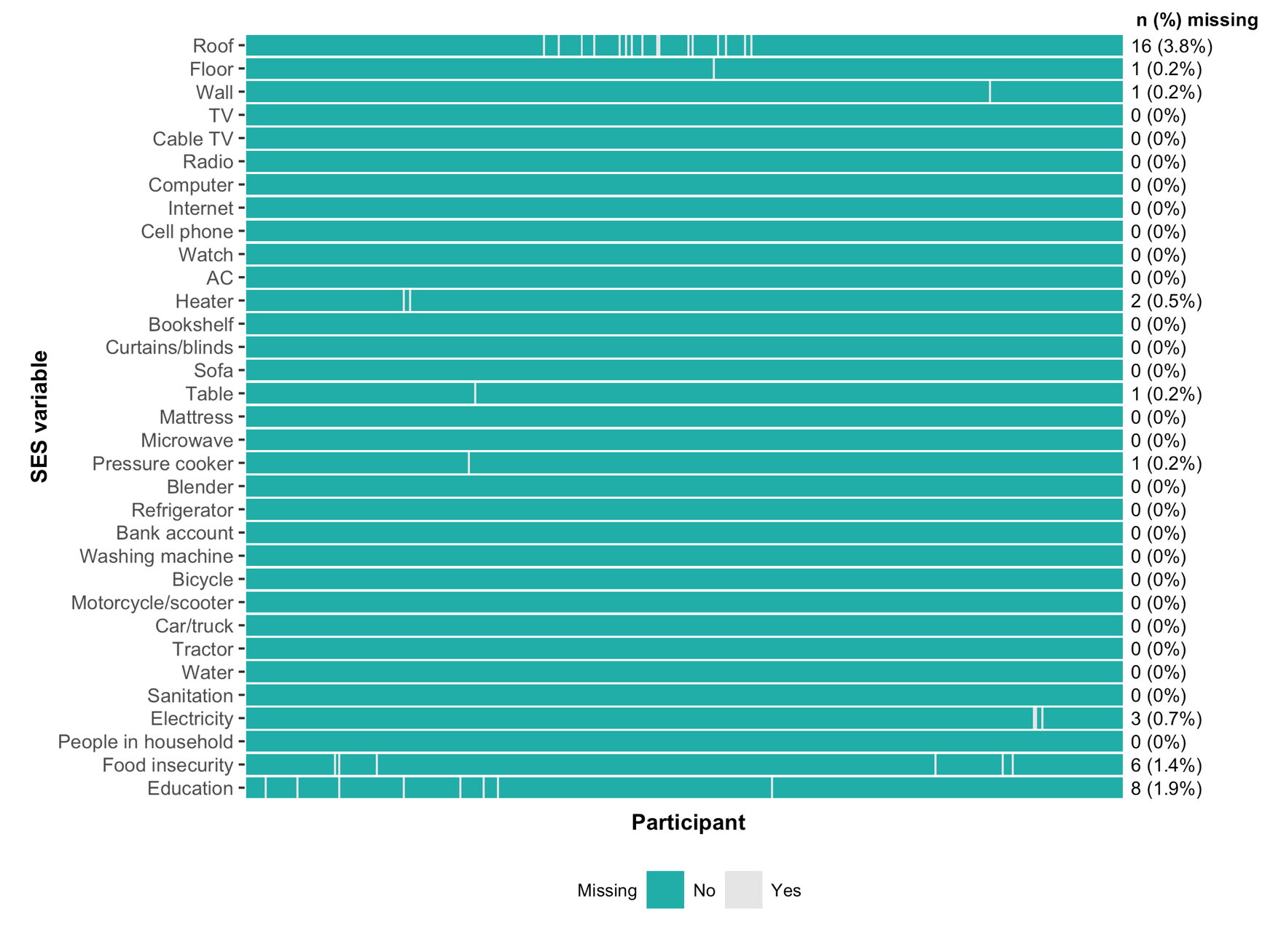
**

**Figure S3: Goodness of fit: Mean and 95% confidence intervals (95% CIs) of expected and observed blood pressure trajectories with age.** Expected values as obtained from the adjusted PM_2.5_, BC and CO generalized additive models for systolic blood pressure (SBP), diastolic blood pressure (DBP), pulse pressure (PP) and mean arterial pressure (MAP). In each panel, the symbols and corresponding vertical lines represent the mean and 95% CIs of the observed blood pressures, respectively, and the orange line and blue shaded area represent the mean and 95% CIs of the expected blood pressures, respectively.


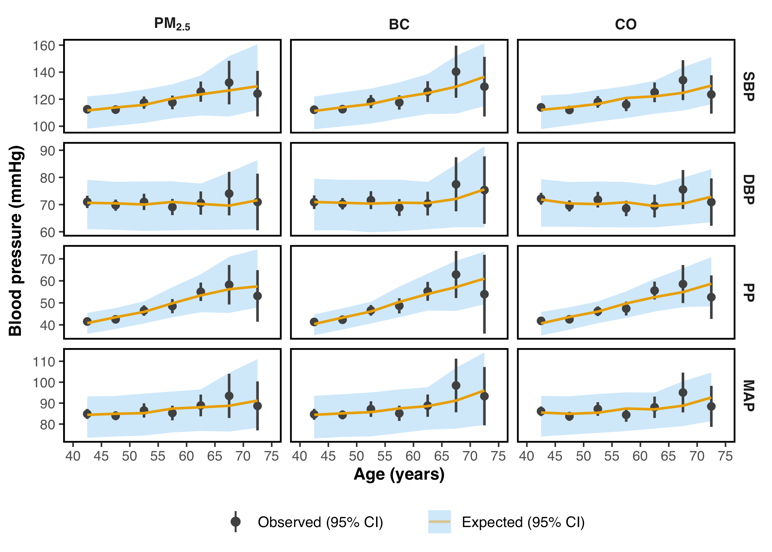


**Figure S4: Goodness of fit: Mean and 95% confidence intervals (95% CIs) of expected and observed blood pressure trajectories with personal exposure to household air pollutants.** Expected values as obtained from the adjusted PM_2.5_, BC and CO generalized additive models for systolic blood pressure (SBP), diastolic blood pressure (DBP), pulse pressure (PP) and mean arterial pressure (MAP). In each panel, the symbols and corresponding vertical lines represent the mean and 95% CIs of the observed blood pressures, respectively, and the orange line and blue shaded area represent the mean and 95% CIs of the expected blood pressures, respectively.


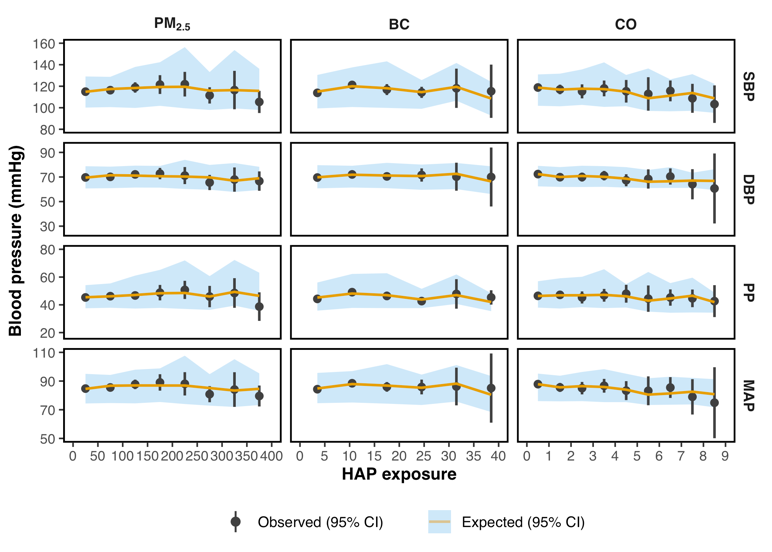


**Figure S5: Missingness plot for baseline variables.** Values displayed on the right represent the number (percentage) missing for each variable.


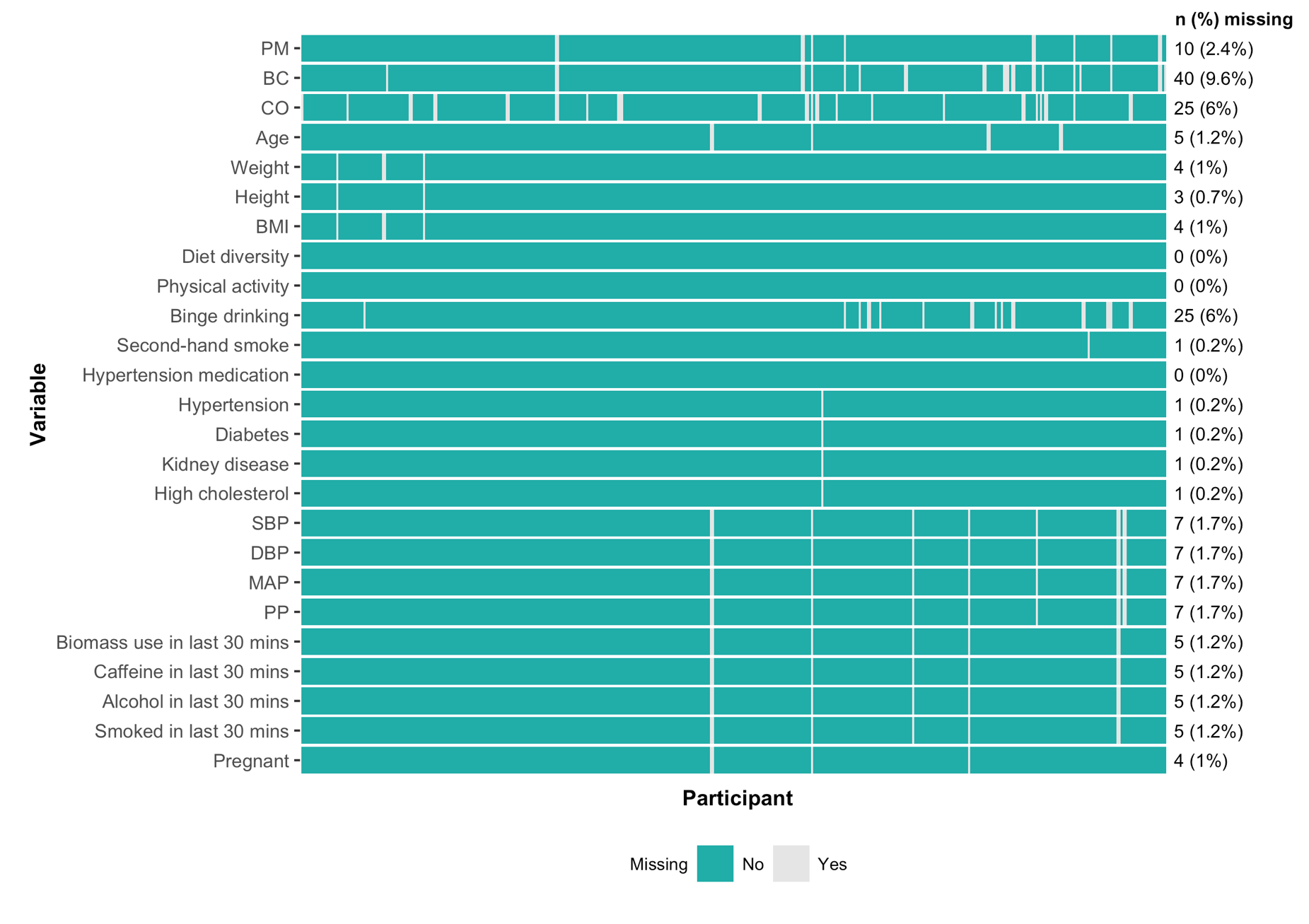


**Figure S6: Distributions of blood pressure measures by site: Cumulative distribution functions and corresponding boxplots of (a) systolic blood pressure, (b) diastolic blood pressure, (c) pulse pressure, and (d) mean arterial pressure.**

**
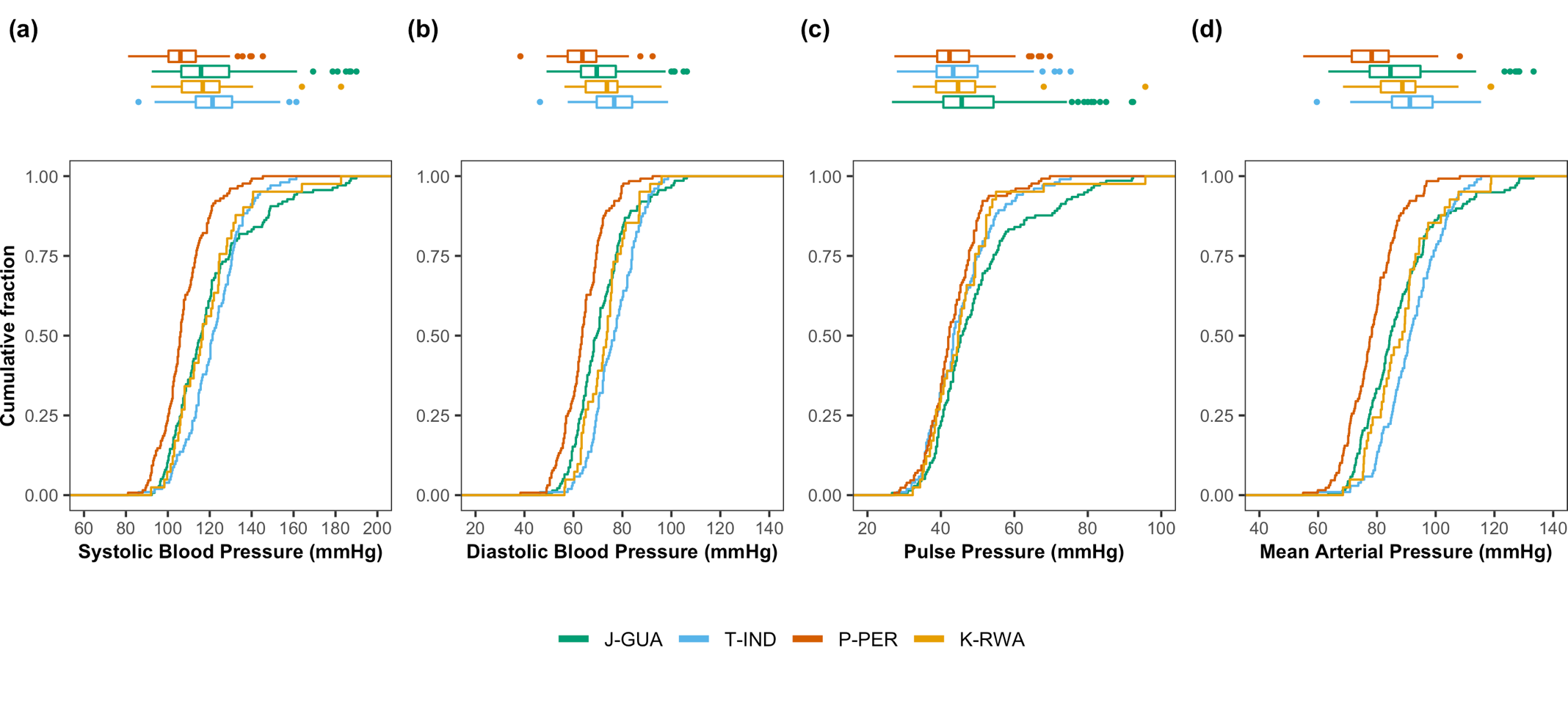
**

**Figure S7: Mean and 95% confidence intervals (95% CIs) of blood pressure measures with age, modeled using natural splines with three degrees of freedom.** (a) Systolic blood pressure (SBP), (b) diastolic blood pressure (DBP), (c) pulse pressure (PP), and (d) mean arterial pressure (MAP).

**
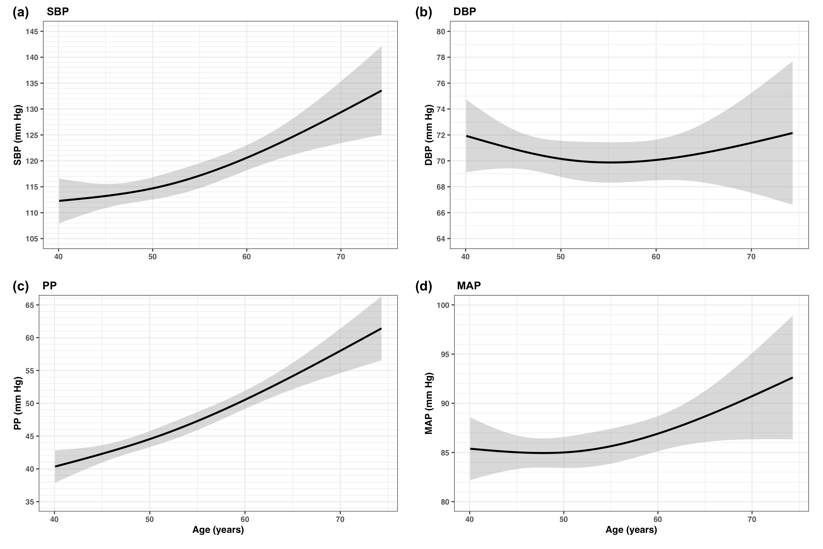
**

**Figure S8: Mean and 95% confidence intervals (95% CIs) of blood pressure measures with body mass index (BMI), modeled using natural splines with three degrees of freedom.** (a) Systolic blood pressure (SBP), (b) diastolic blood pressure (DBP), (c) pulse pressure (PP), and (d) mean arterial pressure (MAP).

**
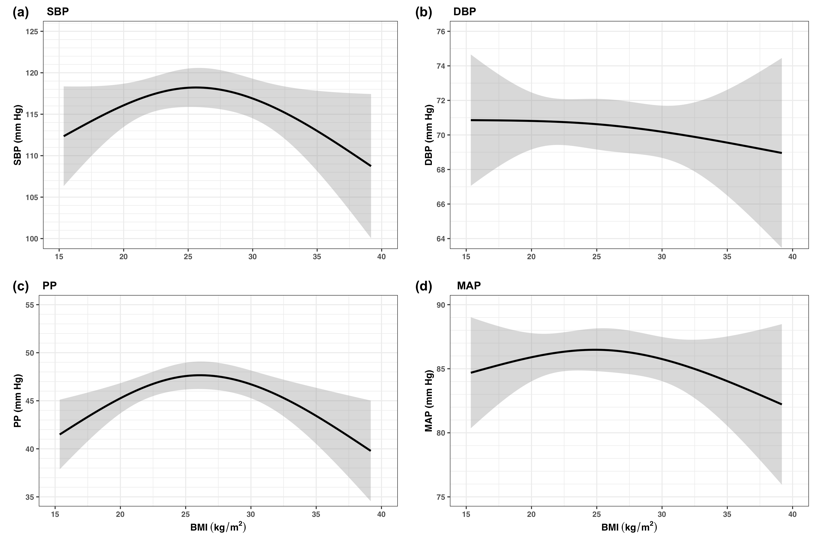
**

**Figure S9: Blood pressure classification of all participants aged 40 – 79 years stratified by site.** The waffle plot shows the number of participants within each BP category where one tile represents one participant, and the table displays the percentage (number) of participants in each BP category. The BP categories from lighter to darker blue are: normal (SBP < 130 mm Hg and DBP < 85 mm Hg), prehypertension (SBP between 130 – 139 mm Hg and/or DBP between 85 – 90 mm Hg), Stage I hypertension (SBP between 140 – 159 mm Hg and/or DBP between 90 – 99 mm Hg), and Stage II hypertension (SBP ≥ 160 mm Hg and/or DBP ≥ 100 mm Hg), based on the 2020 International Society of Hypertension Global Hypertension Practice Guidelines.

**
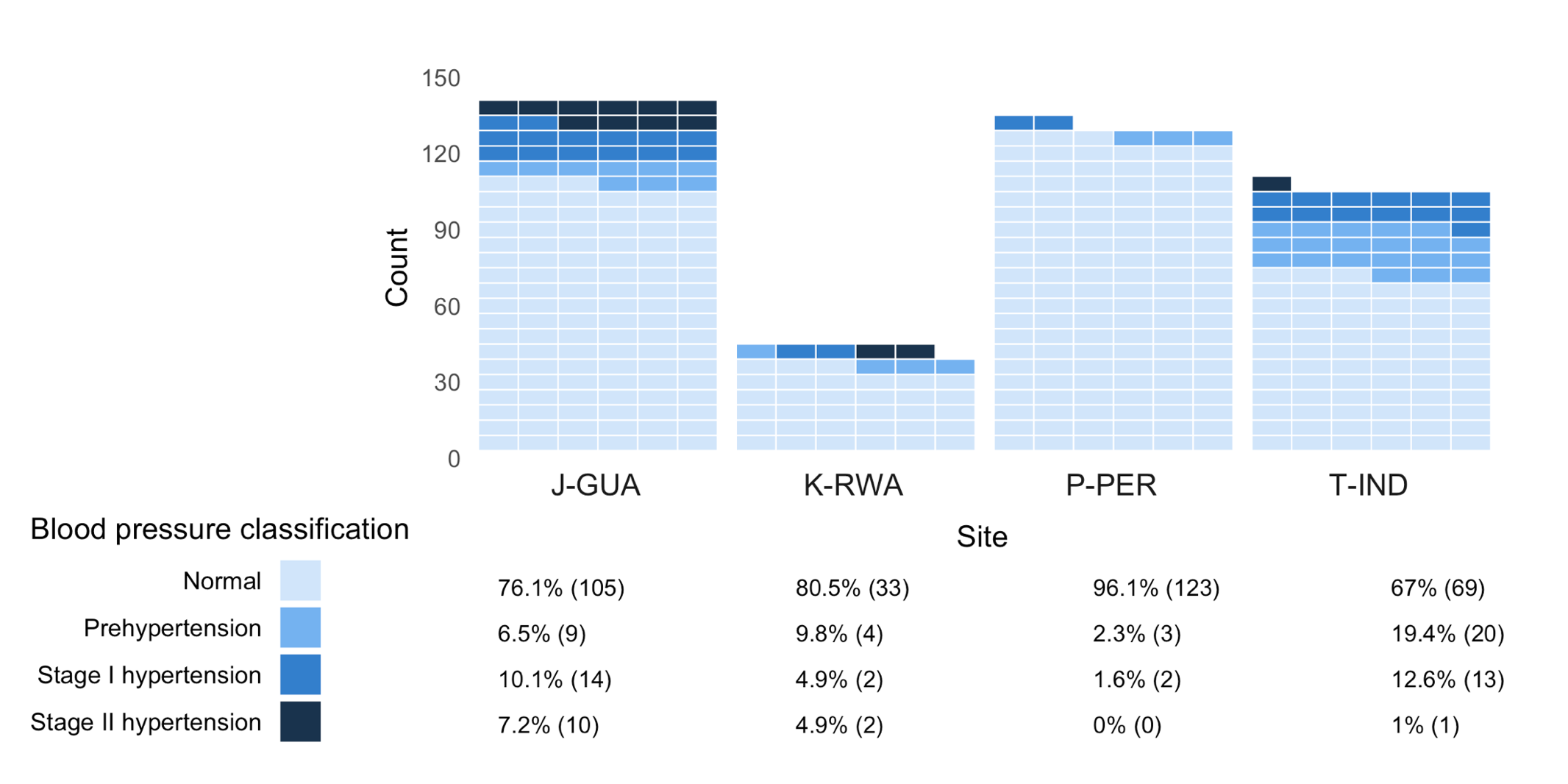
**

**Figure S10: Associations between blood pressure measures and fine particulate matter (PM_2.5_) exposures at 40, 50 and 65 years of age using a generalized additive model of blood pressure as a smooth surface of PM_2.5_ exposures and age adjusted for site and socioeconomic status index in 357 women aged 40-79 years living in resource-poor settings of Tamil Nadu, India; Jalapa, Guatemala; Puno, Peru; and Kayonza, Rwanda. (**a) Systolic blood pressure (SBP), (b) diastolic blood pressure (DBP), (c) pulse pressure (PP), and (d) mean arterial pressure (MAP) at 40 (blue), 50 (green) and 65 (red) years.


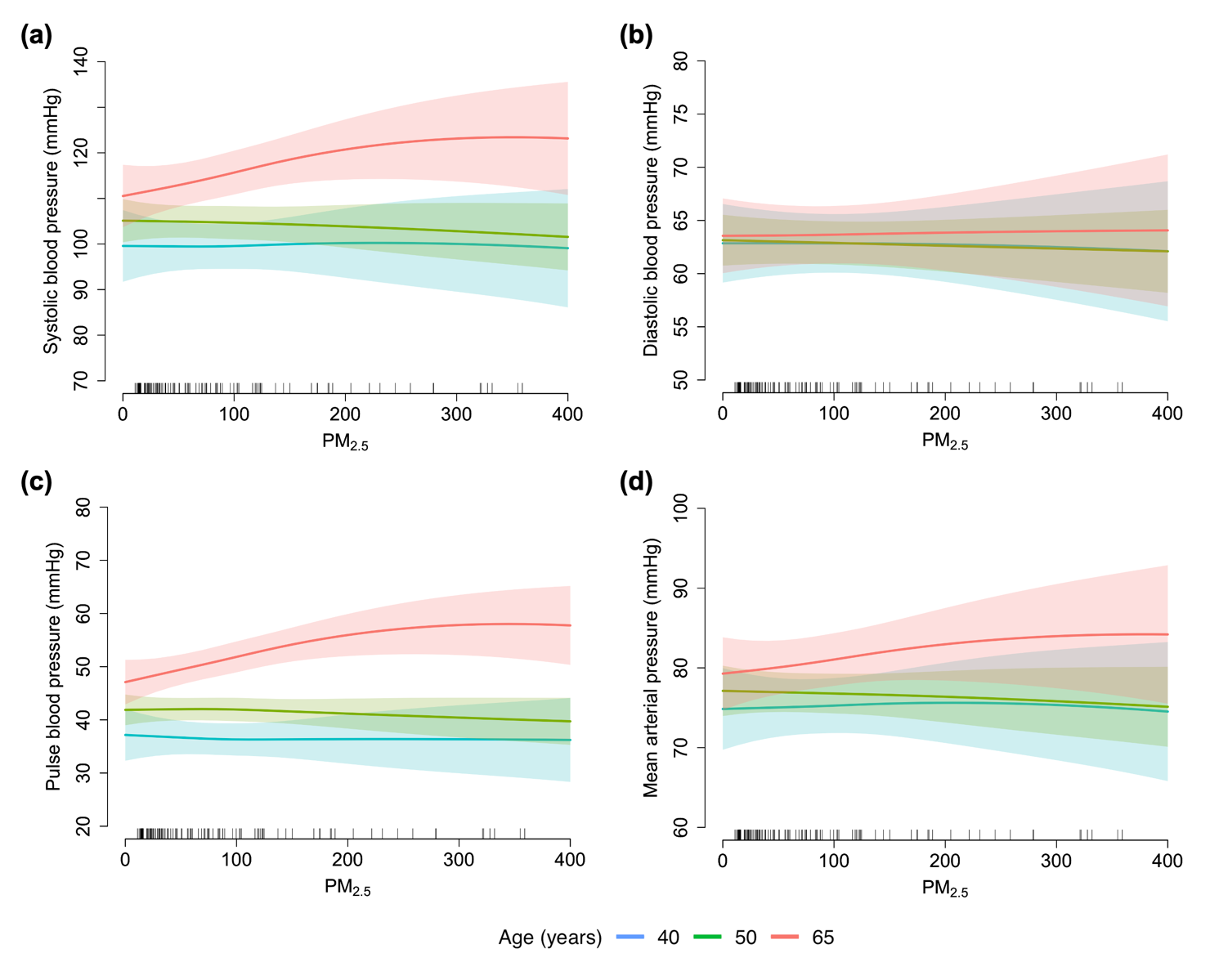


**Figure S11: Estimated mean difference in blood pressure measures between fine particulate matter (PM_2.5_) exposures of 20 to 250 μg/m^3^ and 10 μg/m^3^ at 40, 50 and 65 years of age using a generalized additive model of blood pressure as a smooth surface of PM_2.5_ exposures and age adjusted for site and socioeconomic status index in 357 women aged 40-79 years living in resource-poor settings of Tamil Nadu, India; Jalapa, Guatemala; Puno, Peru; and Kayonza, Rwanda.** Mean differences and 95% pointwise confidence intervals (95% CI) of differences in (a) systolic blood pressure (SBP), (b) diastolic blood pressure (DBP), (c) pulse pressure (PP), and (d) mean arterial pressure (MAP) between PM_2.5_ exposures of 20 to 250 μg/m^3^ and 10 μg/m^3^ at three specific ages: 40, 50 and 65 years. We chose to compare to 10 μg/m^3^ as the lowest interim target (IT-4) recommended by the World Health Organization. In each panel, the diamonds represent the mean differences and the corresponding horizontal lines represent the 95% CIs of the mean differences in blood pressure measures for PM_2.5_ exposures of 20 to 250 μg/m^3^ from blue to red, compared to a PM_2.5_ exposure of 10 μg/m^3^. Values shown in the plots represent concentrations at and above which the mean differences in BP become statistically significant.

**
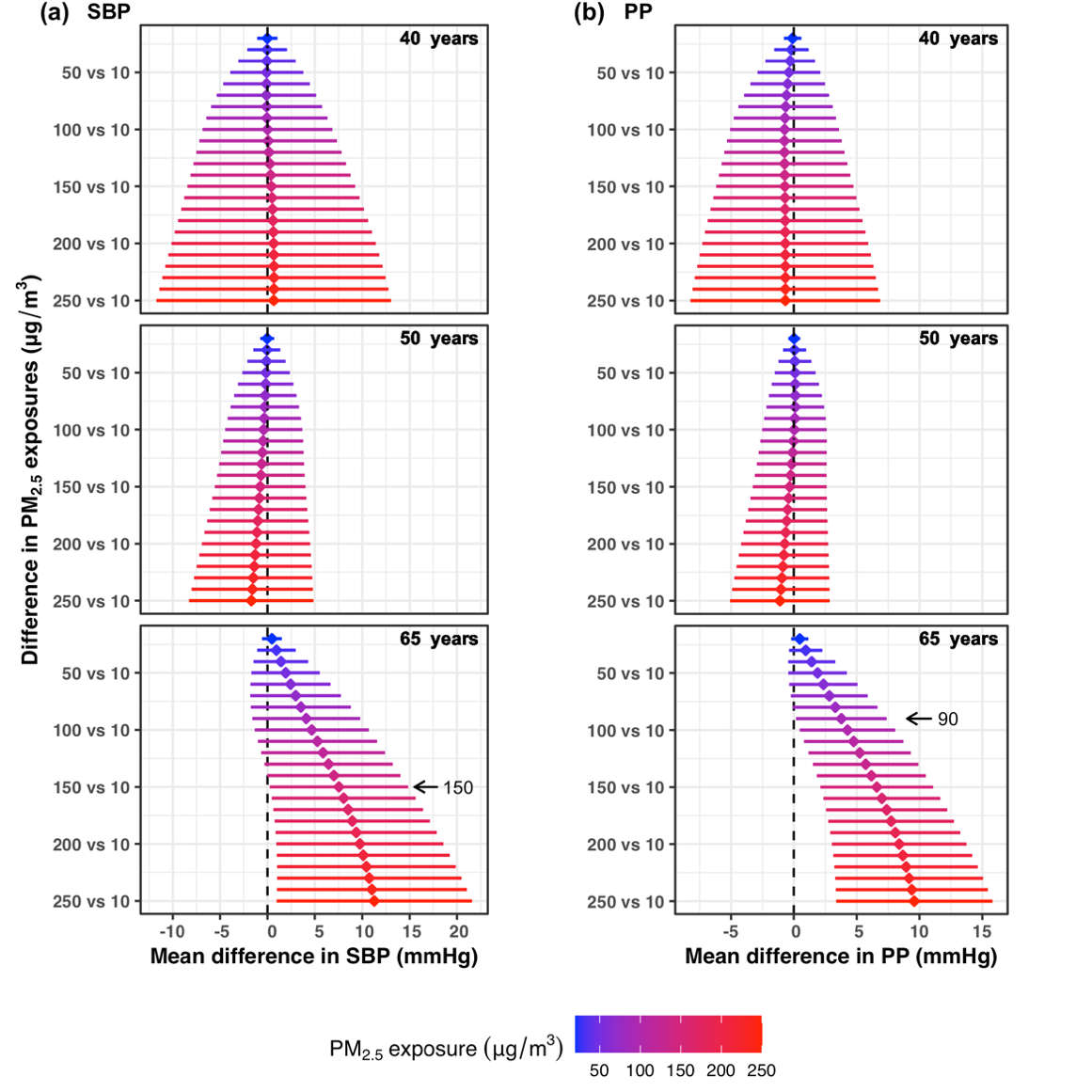
**

**
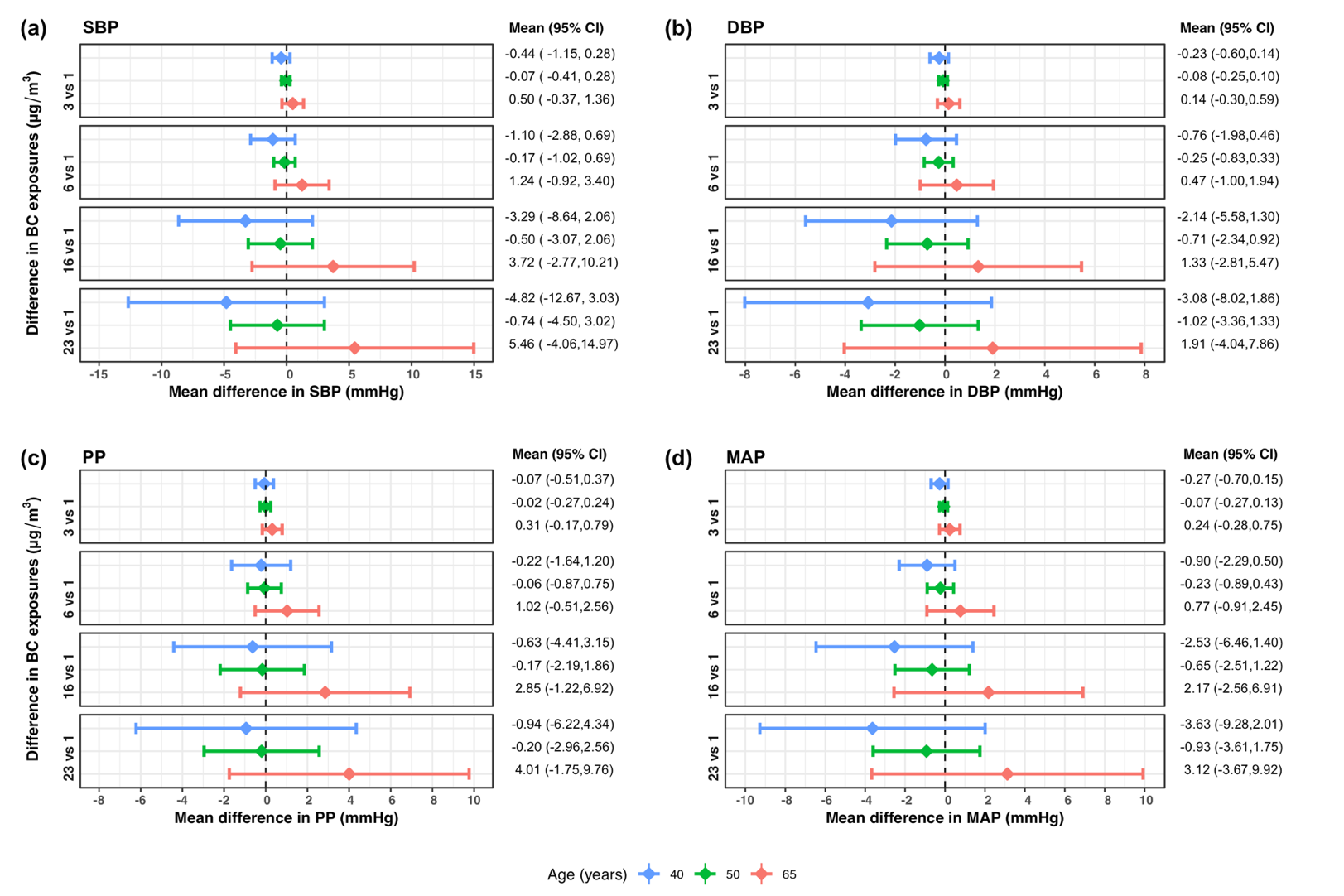
Figure S12: Estimated mean difference in blood pressure measures between black carbon (BC) exposures of either 3, 6, 16 or 23 μg/m^3^ and 1 μg/m^3^ at 40, 50 and 65 years of age using a generalized additive model of blood pressure as a smooth surface of BC exposures and age adjusted for site and socioeconomic status index in 314 women aged 40-79 years living in resource-poor settings of Tamil Nadu, India; Jalapa, Guatemala; Puno, Peru; and Kayonza, Rwanda**. Mean differences and 95% pointwise confidence intervals (95% CI) of differences in (a) systolic blood pressure (SBP), (b) diastolic blood pressure (DBP), (c) pulse pressure (PP), and (d) mean arterial pressure (MAP) between BC exposures of either 3, 6, 16 or 23 μg/m^3^ and 1 μg/m^3^ at three specific ages: 40, 50 and 65 years. These BC exposures were chosen based on the 10^th^, 25^th^, 75^th^ and 90^th^ percentiles. In each panel, the diamonds represent the mean differences and the corresponding horizontal lines represent the 95% CIs of the mean differences at 40 years (in blue), 50 years (in green), and 65 years (in red).

**Figure S13: Estimated mean difference in blood pressure measures between carbon monoxide (CO) exposures of either 0.1, 0.5, 2.9 or 5.8 ppm and 0 ppm at 40, 50 and 65 years of age using a generalized additive model of blood pressure as a smooth surface of BC exposures and age adjusted for site and socioeconomic status index in 362 women aged 40-79 years living in resource-poor settings of Tamil Nadu, India; Jalapa, Guatemala; Puno, Peru; and Kayonza, Rwanda**. Mean differences and 95% pointwise confidence intervals (95% CI) of differences in (a) systolic blood pressure (SBP), (b) diastolic blood pressure (DBP), (c) pulse pressure (PP), and (d) mean arterial pressure (MAP) between CO exposures of either 0.1, 0.5, 2.9 or 5.8 ppm and 0 ppm at three specific ages: 40, 50 and 65 years. These CO exposures were chosen based on the 10^th^, 25^th^, 75^th^ and 90^th^ percentiles. In each panel, the diamonds represent the mean differences and the corresponding horizontal lines represent the 95% CIs of the mean differences at 40 years (in blue), 50 years (in green), and 65 years (in red).

**
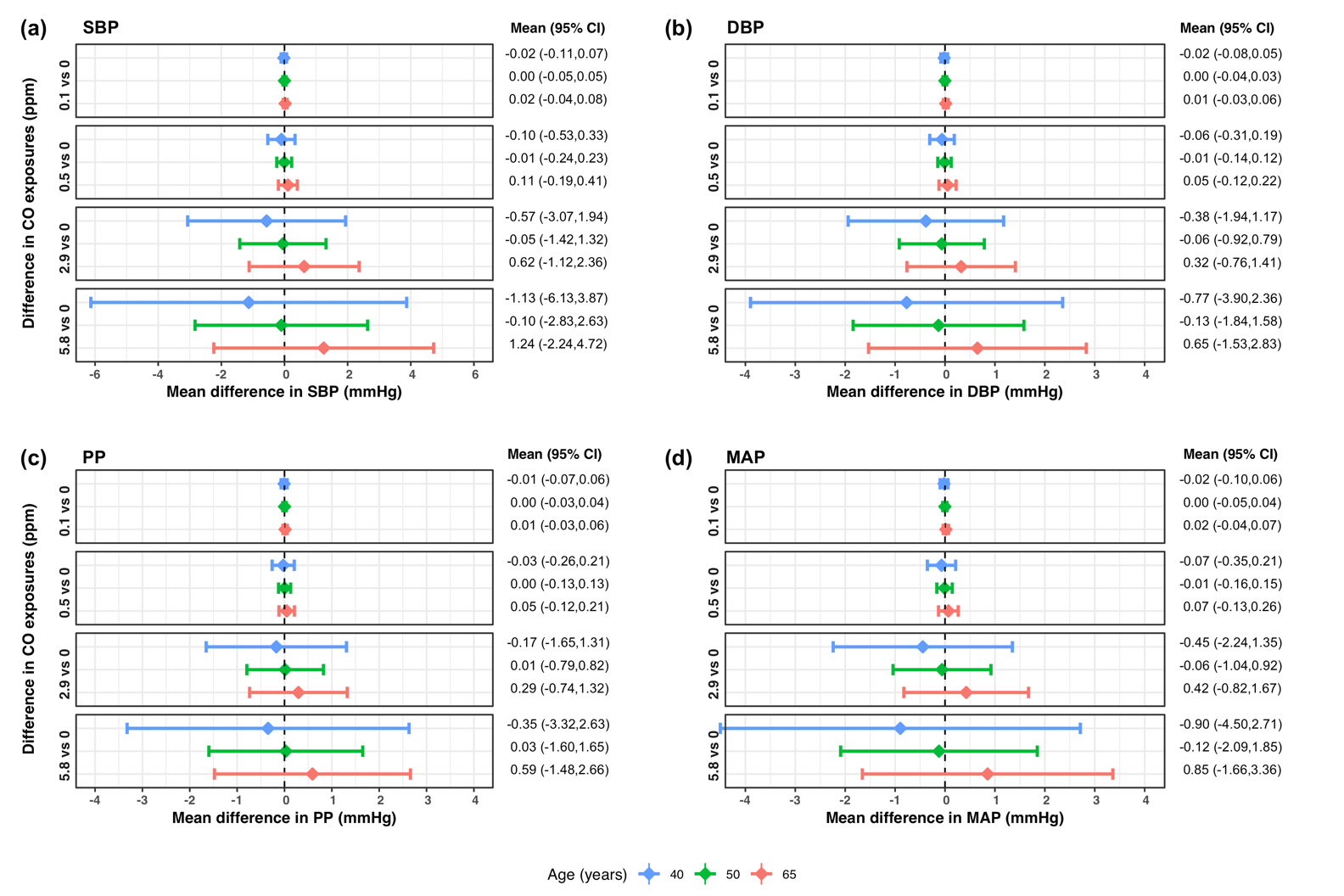
**

**Figure S14: Associations between blood pressure measures and black carbon (BC) exposures at 40, 50 and 65 years of age using a generalized additive model of blood pressure as a smooth surface of BC exposures and age adjusted for site and socioeconomic status index in 314 women aged 40-79 years living in resource-poor settings of Tamil Nadu, India; Jalapa, Guatemala; Puno, Peru; and Kayonza, Rwanda. (**a) Systolic blood pressure (SBP), (b) diastolic blood pressure (DBP), (c) pulse pressure (PP), and (d) mean arterial pressure (MAP) at 40 (blue), 50 (green) and 65 (red) years.


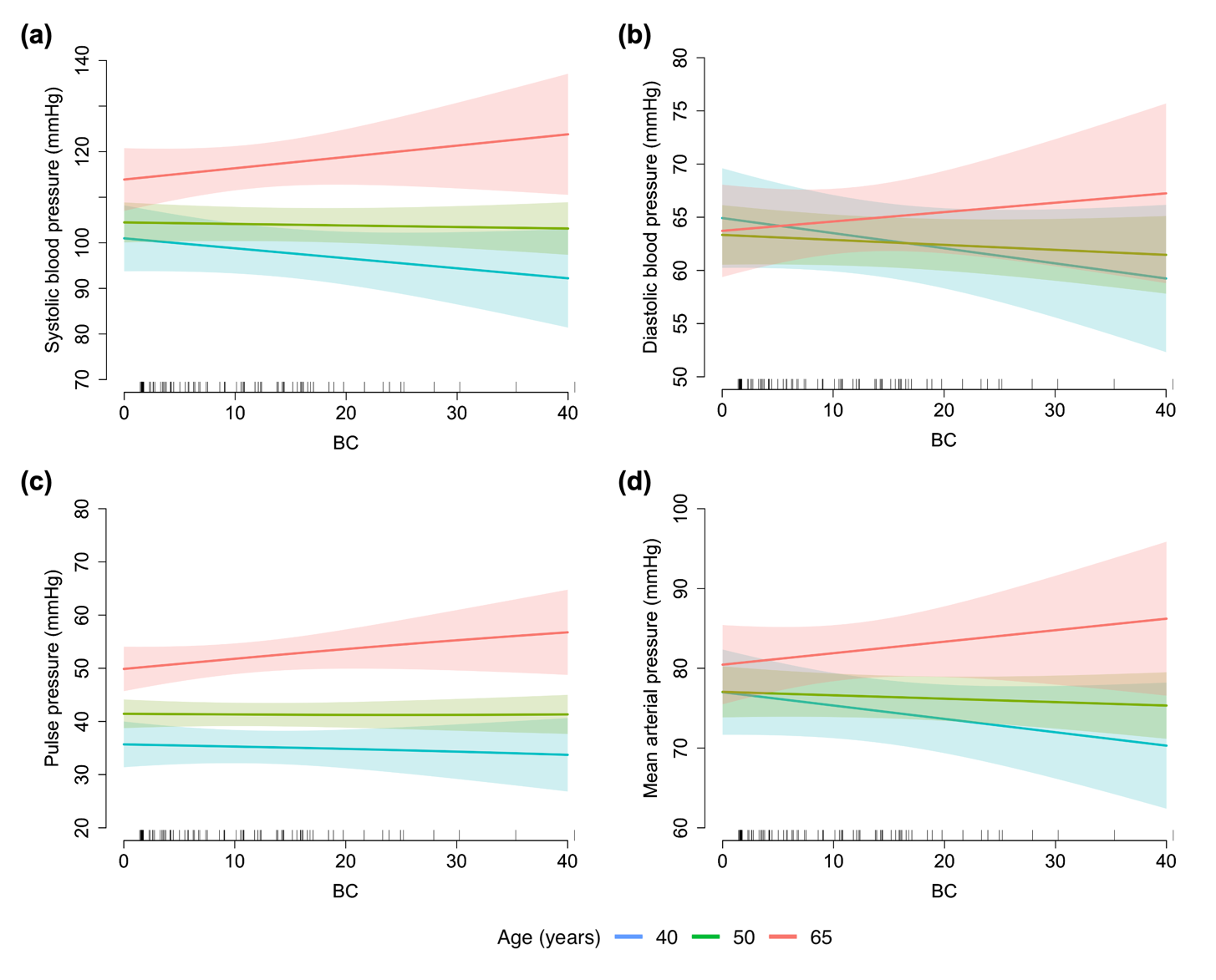


**Figure S15: Associations between blood pressure measures and carbon monoxide (CO) exposures at 40, 50 and 65 years of age using a generalized additive model of blood pressure as a smooth surface of CO exposures and age adjusted for site and socioeconomic status index in 362 women aged 40-79 years living in resource-poor settings of Tamil Nadu, India; Jalapa, Guatemala; Puno, Peru; and Kayonza, Rwanda. (**a) Systolic blood pressure (SBP), (b) diastolic blood pressure (DBP), (c) pulse pressure (PP), and (d) mean arterial pressure (MAP) at 40 (blue), 50 (green) and 65 (red) years.

**
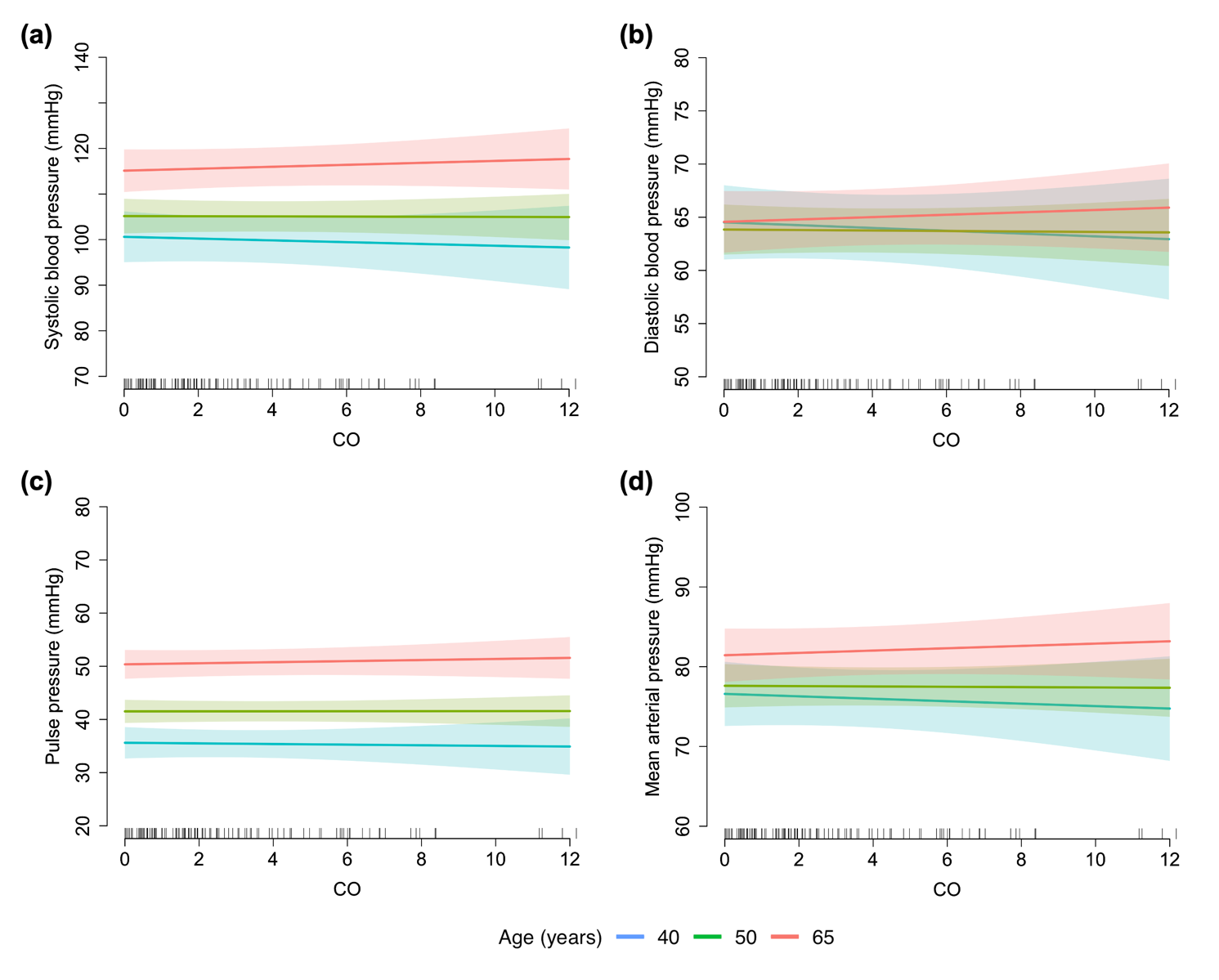
**

**Figure S16: Estimated mean difference in blood pressure measures between fine particulate matter (PM_2.5_) exposures of either 21, 43, 139 or 232 μg/m^3^ and 10 μg/m^3^ for ages <50; 50-64; ≥65 years using a generalized additive model of blood pressure as a spline of PM_2.5_ exposures by age group adjusted for site and socioeconomic status index in 357 women aged 40-79 years living in resource-poor settings of Tamil Nadu, India; Jalapa, Guatemala; Puno, Peru; and Kayonza, Rwanda**. Mean differences and 95% pointwise confidence intervals (95% CI) of differences in (a) systolic blood pressure (SBP), (b) diastolic blood pressure (DBP), (c) pulse pressure (PP), and (d) mean arterial pressure (MAP) between PM_2.5_ exposures of either 21, 43, 139 or 232 μg/m^3^ and 10 μg/m^3^ for ages <50; 50-64; ≥65 years. These PM_2.5_ exposures were chosen based on the 10th, 25th, 75th and 90th percentiles, and 10 μg/m^3^ was chosen as the lowest interim target (IT-4) recommended by the World Health Organization. In each panel, the diamonds represent the mean differences and the corresponding horizontal lines represent the 95% CIs of the mean differences at 40-49 years (in blue), 50-64 years (in green), and ≥65 years (in red).


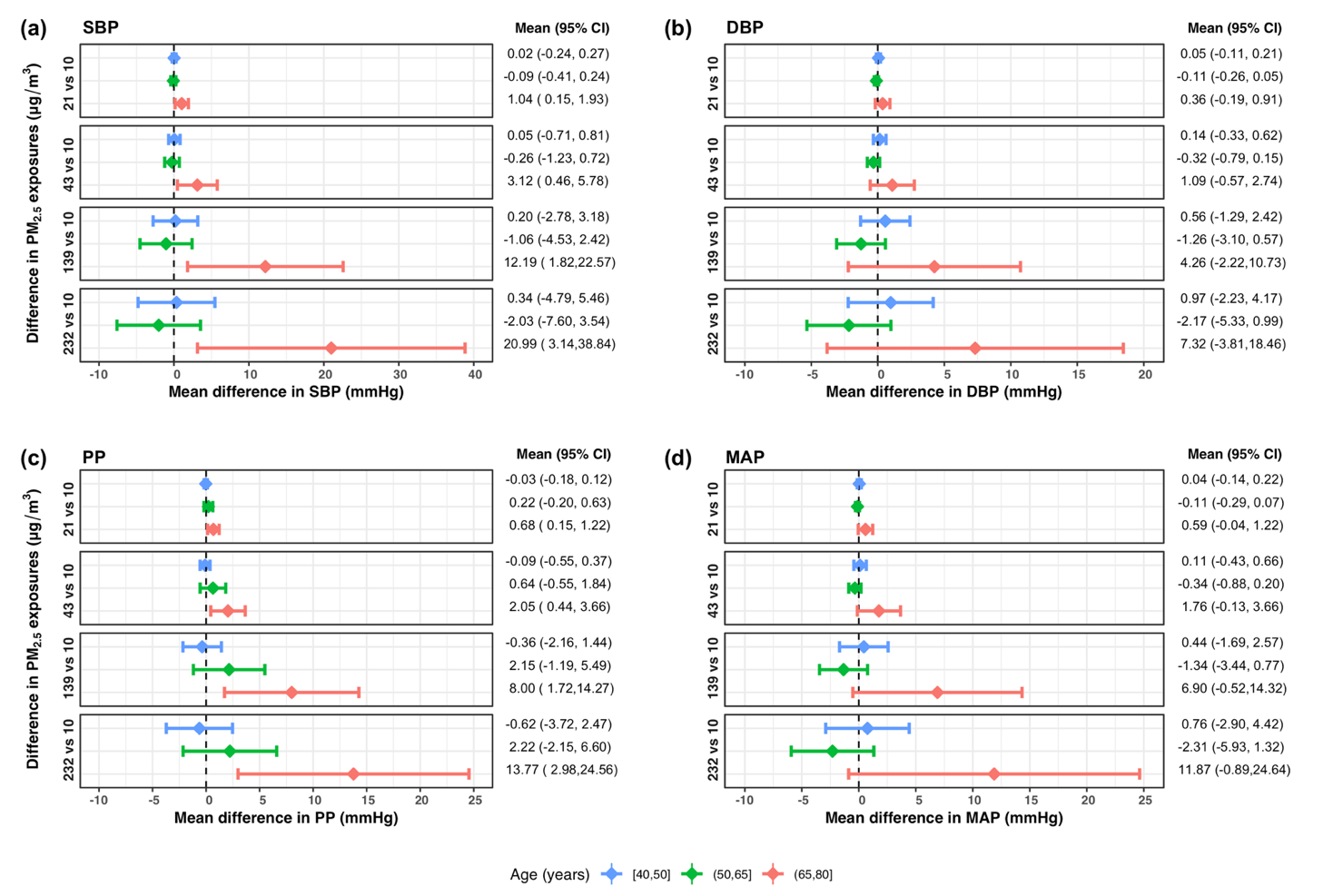


**Figure S17:** **Estimated mean difference in blood pressure measures between black carbon (BC) exposures of either 3, 6, 16 or 23 μg/m^3^ and 1 μg/m^3^ for ages <50; 50-64; ≥65 years using a generalized additive model of blood pressure as a spline of BC exposures by age group adjusted for site and socioeconomic status index in 314 women aged 40-79 years living in resource-poor settings of Tamil Nadu, India; Jalapa, Guatemala; Puno, Peru; and Kayonza, Rwanda.** Mean differences and 95% pointwise confidence intervals (95% CI) of differences in (a) systolic blood pressure (SBP), (b) diastolic blood pressure (DBP), (c) pulse pressure (PP), and (d) mean arterial pressure (MAP) between BC exposures of either 3, 6, 16 or 23 μg/m^3^ and 1 μg/m^3^ for ages <50; 50-64; ≥65 years. These BC exposures were chosen based on the 10^th^, 25^th^, 75^th^ and 90^th^ percentiles. In each panel, the diamonds represent the mean differences and the corresponding horizontal lines represent the 95% CIs of the mean differences at 40-49 years (in blue), 50-64 years (in green), and ≥65 years (in red).

**
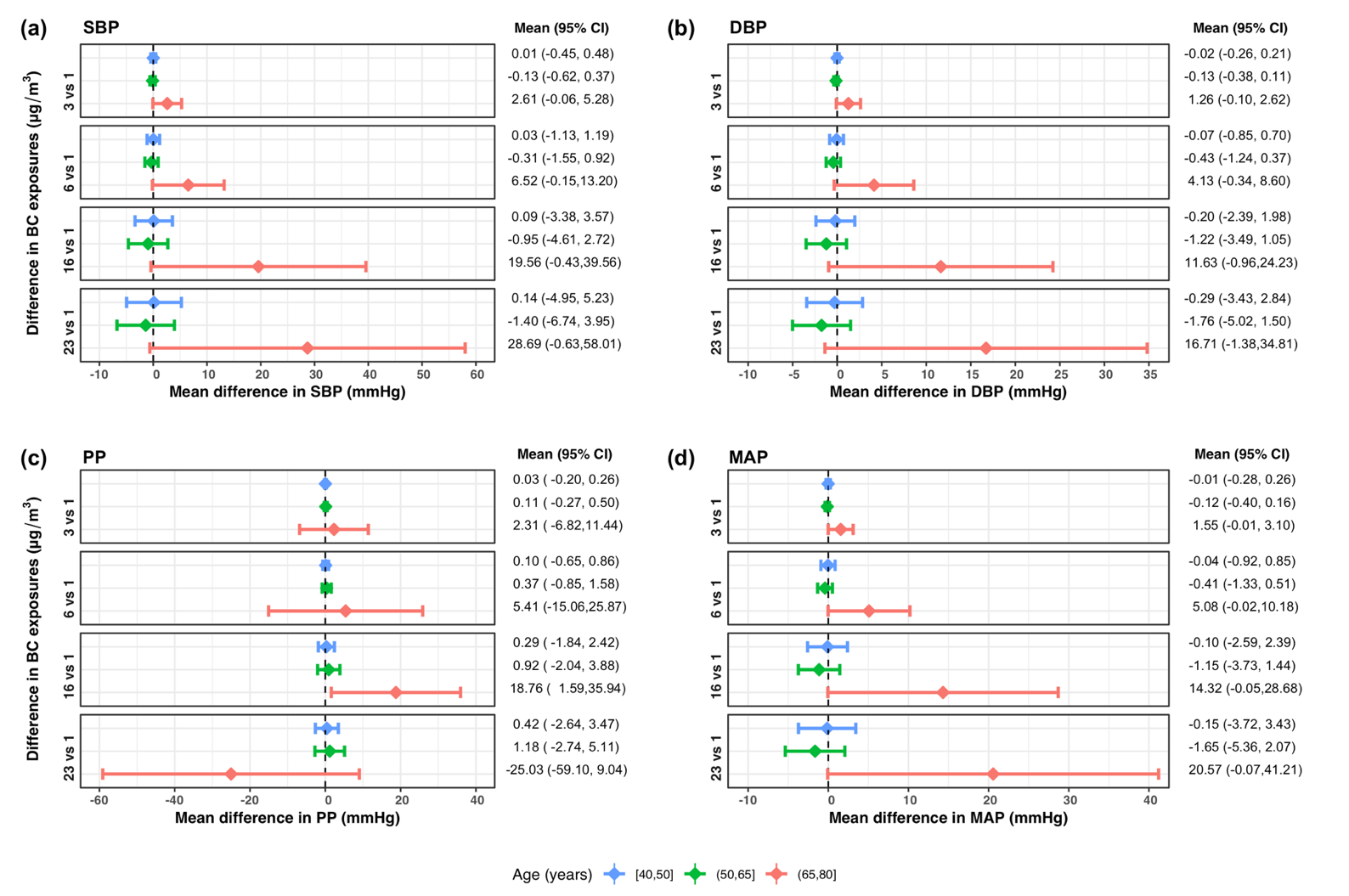
**

**Figure S18: Estimated mean difference in blood pressure measures between carbon monoxide (CO) exposures of either 0.1, 0.5, 2.9 or 5.8 ppm and 0 ppm for ages <50; 50-64; ≥65 years using a generalized additive model of blood pressure as a spline of CO exposures by age group adjusted for site and socioeconomic status index in 362 women aged 40-79 years living in resource-poor settings of Tamil Nadu, India; Jalapa, Guatemala; Puno, Peru; and Kayonza, Rwanda.** Mean differences and 95% pointwise confidence intervals (95% CIs) of differences in (a) systolic blood pressure (SBP), (b) diastolic blood pressure (DBP), (c) pulse pressure (PP), and (d) mean arterial pressure (MAP) between CO exposures of either 0.1, 0.5, 2.9 or 5.8 ppm and 0 ppm for ages <50; 50-64; ≥65 years. These CO exposures were chosen based on the 10^th^, 25^th^, 75^th^ and 90^th^ percentiles. In each panel, the diamonds represent the mean differences and the corresponding horizontal lines represent the 95% CIs of the mean differences at 40-49 years (in blue), 50-64 years (in green), and ≥65 years (in red).


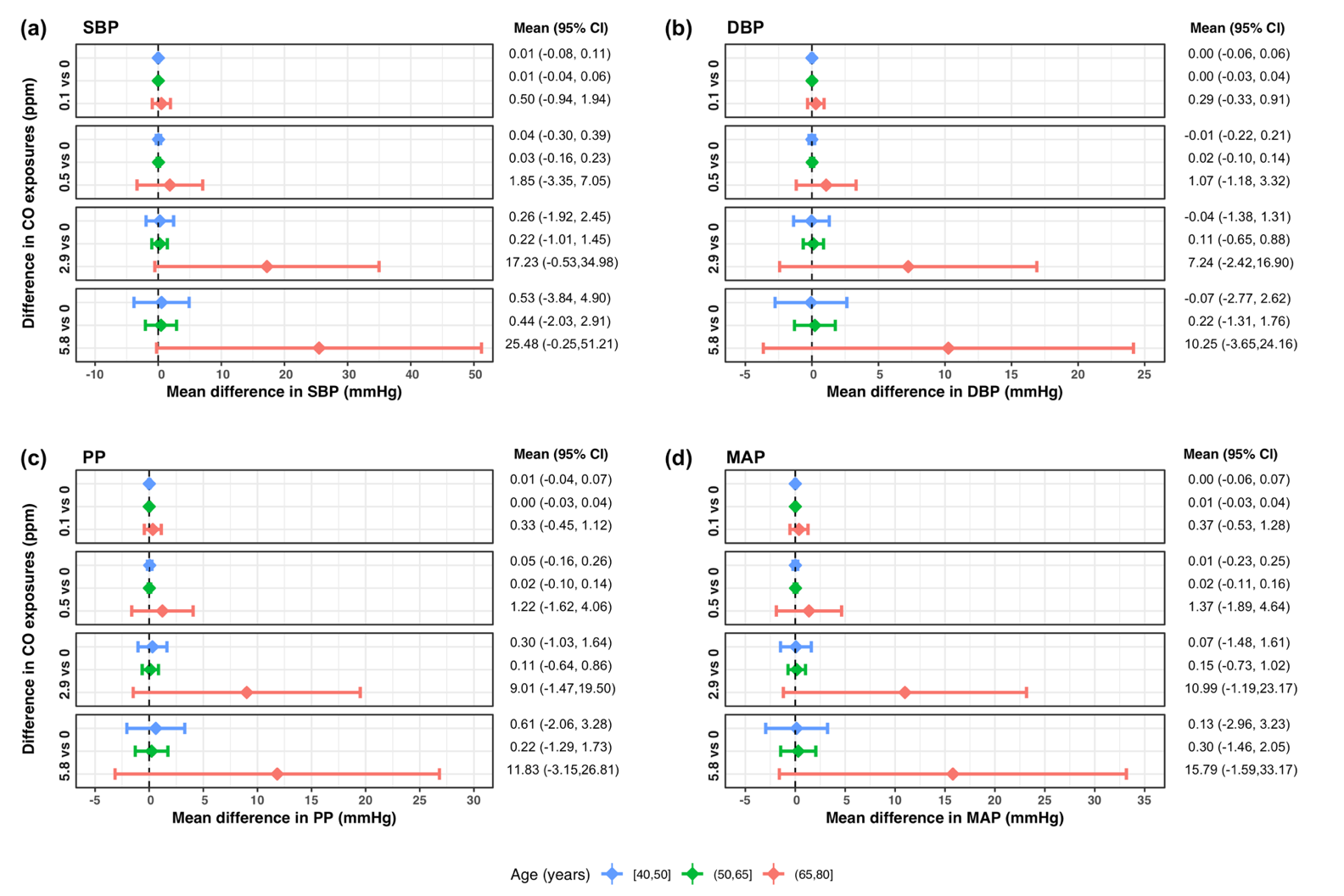


**Figure S19: Associations between blood pressure measures and fine particulate matter (PM_2.5_) exposures at 65 years of age for BMIs of 22, 25 and 30 kg/m^2^ using a generalized additive model of blood pressure as a smooth surface of PM_2.5_ exposures, age and BMI, adjusted for site and socioeconomic status index in 357 women aged 40-79 years living in resource-poor settings of Tamil Nadu, India; Jalapa, Guatemala; Puno, Peru; and Kayonza, Rwanda. (**a) Systolic blood pressure (SBP), (b) diastolic blood pressure (DBP), (c) pulse pressure (PP), and (d) mean arterial pressure (MAP) at 22 (blue), 25 (green) and 30 (red) kg/m^2^.
